# A Comparative Analysis of Ash Leaf-Colonizing Bacterial Communities Identifies Putative Antagonists of *Hymenoscyphus fraxineus*

**DOI:** 10.3389/fmicb.2020.00966

**Published:** 2020-05-29

**Authors:** Kristina Ulrich, Regina Becker, Undine Behrendt, Michael Kube, Andreas Ulrich

**Affiliations:** ^1^Institute of Forest Genetics, Johann Heinrich von Thünen Institute, Waldsieversdorf, Germany; ^2^Microbial Biogeochemistry, Research Area Landscape Functioning, Leibniz Centre for Agricultural Landscape Research (ZALF), Müncheberg, Germany; ^3^Integrative Infection Biology Crops-Livestock, University of Hohenheim, Stuttgart, Germany

**Keywords:** ash dieback, *Fraxinus excelsior*, microbiota, antagonism, dual cultures, healthy core microbiome, phyllosphere

## Abstract

In the last few years, the alarming spread of *Hymenoscyphus fraxineus*, the causal agent of ash dieback, has resulted in a substantial threat to native ash stands in central and northern Europe. Since leaves and leaf petioles are the primary infection sites, phyllosphere microorganisms are presumed to interact with the pathogen and are discussed as a source of biocontrol agents. We studied compound leaves from susceptible and visible infection-free trees in four ash stands with a high likelihood of infection to assess a possible variation in the bacterial microbiota, depending on the health status of the trees. The bacterial community was analyzed by culture-independent 16S rRNA gene amplicon sequencing and through the isolation and taxonomic classification of 2,589 isolates using matrix-assisted laser desorption/ionization time-of-flight mass spectrometry (MALDI-TOF MS). The bacterial community structure did not show significant differences. However, a set of amplicon sequence variants (ASVs) and MALDI groups belonging to *Luteimonas*, *Aureimonas*, *Pseudomonas*, *Bacillus*, and *Paenibacillus* were distinctly increased in tolerant trees, which may be associated with the ability of the tree to resist the pathogen. The most obvious differences were observed for *Luteimonas*, a genus that is also exclusively present in the healthy core microbiome. In a first *in vitro* screen of antagonists, approximately 11% of total isolates suppressed the growth of *H. fraxineus*, but a statistical test with two different *H. fraxineus* strains confirmed only the antagonistic activity of 8% of these isolates. The antagonistic isolates were assigned to *Bacillus velezensis*, *Pantoea vagans*, and *Pseudomonas caspiana.* Overall, our study provides a set of isolates or phylogenetic groups that might be involved in the process that prevents the penetration and spread of *H. fraxineus.* In the next step, *in planta* experiments are required with a longer period of exposure to *H. fraxineus* to evaluate effective isolates or consortia of isolates acting through direct antagonism or competition or indirectly by inducing resistance.

## Introduction

Starting in northeastern Poland in the early 1990s, the severe dieback of common ash (*Fraxinus excelsior*) with a high mortality rate has spread across the European continent and is now present almost throughout the entire natural distribution range of European ash (Kowalski and Holdenrieder, 2009a; McKinney et al., 2014; Vasaitis and Enderle, 2017). The causal agent of ash dieback is the invasive ascomycete fungus *Hymenoscyphus fraxineus* (T. Kowalski) (Baral et al., 2014). This pathogen is considered to originate from Asia, where it is reported to have an asymptotic association with *Fraxinus mandshurica* and *Fraxinus chinensis* ssp. *rhynchophylla* (Zhao et al., 2013; Cleary et al., 2016). In Europe, it is killing ash at an alarming rate and displacing the non-aggressive indigenous fungus *Hymenoscyphus albidus* (Drenkhan et al., 2017).

Less than 5% of trees are partially resistant or tolerant to ash dieback disease, and trees of all ages are affected at various site types in forest, urban, and nursery settings (Timmermann et al., 2011; McKinney et al., 2014). While young trees often die within a few years after infection, older trees generally become chronically diseased and more susceptible to root rot diseases caused by *Armillaria* spp. (Skovsgaard et al., 2010; Langer et al., 2015; Chandelier et al., 2016).

The infection of leaves by *H. fraxineus* derives from the windborne ascospores produced between June and September in sexual fruiting bodies (apothecia) that are mainly growing on the petioles of ash leaves that have fallen from infected trees after overwintering on soil debris (Queloz et al., 2011). *H. fraxineus* behaves as a necrotrophic pathogen. The ascospores germinate on the leaf surface and leaf petioles. After penetrating the cuticle and the epidermis by forming appressoria, the fungus causes dark brown/orange lesions on leaflets, which then expand proximally and spread into petioles (Hanackova et al., 2017b). After crossing the petiole–shoot junction, the pathogen may also spread into shoots and twigs, causing necrotic bark lesions. Xylem vessels are occluded, and as branches are girdled by lesions in the cambium, the crown starts dying back. However, shoot infection is the endpoint in the life cycle of *H. fraxineus* because fructification only very rarely occurs on dead stems (Gross et al., 2014). The loss of leaves in the crown of mature trees proceeds over several years and leads to tree death in severe cases (Cleary et al., 2013; Gross et al., 2014; McKinney et al., 2014). The fungus overwinters among the leaf litter, where it undergoes an extensive saprophytic stage. In early summer, new apothecia appear on the pseudosclerotial leaf rachises, mostly on petioles from the previous year (Kowalski and Holdenrieder, 2009b; Gross et al., 2014) and fire ascospores up into the air (100,000 ascospores per cubic meter were found in infected areas). These ascospores are distributed widely by the wind (Hietala et al., 2013; Chandelier et al., 2014) and carried to ash leaves to complete the life cycle (Gross et al., 2012).

Since leaves and leaf petioles are the primary infection sites for establishing new infections on trees (Cleary et al., 2013; Schwanda and Kirisits, 2016) and *H. fraxineus* is able to complete its entire life cycle on ash leaves, the pathogen might be hampered by bacteria and fungi colonizing leaf surfaces or living inside the leaves (Cross et al., 2017; Hanackova et al., 2017a; Schlegel et al., 2018).

Several studies have explored the composition of fungal communities in *F. excelsior* leaves and shoots (Unterseher et al., 2007; Davydenko et al., 2013; Kosawang et al., 2018; Schlegel et al., 2018). Seasonal, site-specific, and vertical changes were observed in the identified fungal communities (Scholtysik et al., 2013; Cross et al., 2017; Hanackova et al., 2017a), but distinct differences in the fungal communities on leaves of *F. excelsior* trees with and without dieback symptoms were not detected (Griffiths et al., 2020). According to Schlegel et al. (2016), ash leaf-inhabiting fungal endophytes produce antifungal secondary metabolites that inhibit the germination of *H. fraxineus* ascospores *ex situ*. Furthermore, fungal endophytes with a high antagonistic activity against *H. fraxineus in vitro* and the potential to serve as biological control agents against ash dieback were isolated (Junker, 2013; Schulz et al., 2015; Hanackova et al., 2017a; Kosawang et al., 2018).

Although communities of fungal endophytes and epiphytes inhabiting *F. excelsior* leaves are well studied, very little is known about the bacterial community of common ash and how the bacteria interact with the pathogen *H. fraxineus*. Recently, Griffiths et al. (2020) analyzed the bacterial community structure of *F. excelsior* leaves in the United Kingdom but did not observe a significant effect of the *H. fraxineus* infection. They identified a small proportion of genera negatively correlated with the intensity of the *H. fraxineus* infection. However, studies of culturable bacteria and their antagonistic potential toward *H. fraxineus* are still lacking.

Bacteria directly attack fungal pathogens by secreting antibiotic compounds, including heterocyclic phenazines (Mavrodi et al., 2006) and cyclic lipopeptides (Ongena et al., 2007) or hydrogen cyanide as a volatile compound that acts directly on cells by blocking cytochrome c oxidase. The production of extracellular hydrolytic enzymes has an important role in degrading the cell wall polymers of fungal plant pathogens (Wu et al., 2018). However, the fungal pathogen is also suppressed through competitions for resources such as nutrients or space (e.g., by siderophores), niche colonization, blockage of the potential entry points of the pathogen, or prevention of the germination of its propagules, as well as indirectly through the induction of systemic resistance (Terhonen et al., 2018; Schlechter et al., 2019). Due to their protective effects, plant-associated bacterial isolates represent potential biocontrol agents in a number of trees (Brooks et al., 1994; Barriuso et al., 2008; Melnick et al., 2011; Prieto et al., 2011). In the last few years, research examining the correlation between bacteria and fungi with plant health has shifted from single strains or species to a more community-based view that focuses on microbiomes (Berg et al., 2017; Köberl et al., 2017; Witzell and Martin, 2018). Koskella et al. (2017) reported a link between the community composition of bark-associated bacteria and symptoms of bleeding canker in *Castanea dentata*, suggesting that the tree microbiota affected the spread of the disease.

In addition to stochastic colonization, active recruitment of useful microbial communities (Muller et al., 2016; Lemanceau et al., 2017; Hamonts et al., 2018) is presumed to be an operational mechanism for shaping the microbiota of long-living trees (Witzell and Martin, 2018). Other factors are also known to modulate the plant microbiota, including local conditions, plant age and fitness, climate, and pathogen presence (Compant et al., 2019). Accordingly, bacteria specific for the leaves of tolerant ash trees in severely affected stands may be associated with the ability of the tree to resist the pathogen or are potentially involved in protecting the host from ash dieback. Therefore, in this study, ash trees with visible symptoms of ash dieback were compared with healthy-looking trees, which were recorded as having a relatively high tolerance to *H. fraxineus*. We hypothesize that significant differences in the bacterial leaf microbiome exist, and the comparison of these trees will reveal bacteria that are capable of suppressing the pathogen. A high-throughput sequencing approach is combined with cultivation methods to analyze the microbiome as completely as possible and to overcome the biases of both procedures. We further assume that plant-associated bacteria adapted to ash trees are a reservoir for isolates with antagonistic potential. The ability to directly attack the fungal pathogen is tested *in vitro* by performing dual-culture assays.

## Materials and Methods

### Study Site and Sampling Strategies

Field sampling was conducted in July 2017 at four ash forest districts in Northeast Germany with a severe infestation of *H. fraxineus* infection: Lendershagen (54°23′N, 12°83′E; plot A), Karnin (54°26′N, 12°87′E; plot B), Pennin (54°25′N, 13°E; plot C), and Wredenhagen (53°30′N, 12°58′E; plot D). In each of the districts, four pairs of adjacent trees consisting of a susceptible and a tolerant individual (age between 60 and 80 years) were selected. The distance between the tree pairs within the districts was at least 80 m. Healthy trees were very rarely observed in the long-term monitoring plots and were previously recorded as having relatively high tolerance to ash dieback because they had grown in a site where the pathogen has been present for several years (Sündermann and Jütte, 2014). Compound leaves with petioles from healthy trees were sampled by shooting down branches using a catapult; diseased trees were cut down to sample the leaves. Representative samples were collected from the middle part of the crowns from both healthy and susceptible trees. To exclude the effect of the pathogen *H. fraxineus* on the indigenous microbial community, visually healthy leaves and petioles were sampled from both susceptible and tolerant trees. Altogether, samples from 32 trees were collected and stored in plastic bags at 4°C for transportation and processed within 24 h.

In the laboratory, leaves, leaflets, petioles, and rachis were randomly chosen from compound ash leaves. An aliquot of the samples (4 g) was ground in 4 ml of sterile 0.85% NaCl with a sterile mortar and pestle and used for cultivation. Another aliquot of the samples (4 g) was ground in liquid nitrogen and stored at −80°C until DNA extraction.

### DNA Extraction and Amplicon Sequencing From Ash Leaves

Total DNA was extracted from 100 mg of the ground plant material using the DNeasy Plant Mini Kit (Qiagen, Hilden, Germany). The cells were disrupted again with the FastPrep-24 Instrument (MP Biomedicals, Germany; 30 s at 6 ms^–1^), and extraction was performed using a standard protocol. The degree of DNA degradation was monitored on 1% agarose gels. The DNA purity and concentration were measured using a NanoDrop spectrophotometer. The V5–V6 region of the 16S rRNA genes was amplified using the primers 799F and 1115R, which exclude the chloroplast and mitochondria DNA of the host plant (Chelius and Triplett, 2001; Redford et al., 2010). The primers contained a heterogeneity spacer along with a barcode sequence. The amplicons were purified with a MSB Spin PCRapace kit (Invitek, Berlin, Germany) and mixed at equimolar DNA concentrations. Library preparation and Illumina MiSeq 300-bp paired-end sequencing was performed at LGC Genomics (Berlin, Germany).

### Data Processing and Statistical Analysis

The raw sequence data were processed with the DADA2 R package v. 1.12.1 (Callahan et al., 2017) and mothur v. 1.39.1 (Schloss et al., 2009; RRID: SCR_011947). The DADA2 algorithm was applied for quality filtering and removing chimeric sequences. Based on a parametric error model, the pipeline provides amplicon sequence variants (ASVs), in contrast to the traditional operational taxonomic unit (OTU) approach, and reduces the problem of falsely identified OTUs originating from mis-clustered sequences (Fricker et al., 2019). The ASVs were taxonomically assigned by the naive Bayesian classifier method using the Silva reference database v. 132 (RRID: SCR_006423). In addition, the assignment was completed at the species level if the ASVs exactly matched the sequence of the reference strain of only one species. The taxonomic assignment was confirmed with the taxonomic classification in mothur using the RDP training set 16 (RRID: SCR_006633) and the Silva database. Statistical analyses were performed using the phyloseq, vegan, and ape packages of R 3.6.0, as well as MicrobiomeAnalyst (Chong et al., 2020; RRID: SCR_015022). All samples were rarefied to the minimum number of sequences among all samples. Three diversity indices (Chao 1, Shannon, and Simpson) were calculated. The community structure was compared by applying a principal coordinate analysis (PCoA) based on an unweighted UniFraq distance matrix of the ASVs. Significant differences between the bacterial communities were tested using analysis of similarity (ANOSIM). A taxonomic network was constructed using Cytoscape version 3.7.1 to visualize differences in the core microbiome (Shannon et al., 2003; RRID: SCR_003032). The differential abundance at different taxonomic levels was analyzed with the metagenomeSeq tool using the false discovery rate (FDR) for multiple test correction (Paulson et al., 2013).

The paired sequence reads were deposited in the public repository Sequence Read Archive (SRA, RRID: SCR_004891) with the accession number PRJNA602193.

### Isolation of Bacteria

For the isolation of epiphytic and endophytic bacteria, the ground plant material from each sample was serially diluted in 0.85% NaCl and plated on R2A, a nutrient-poor medium that is suitable for the growth of diverse plant-associated bacteria (Difco, Detroit, United States). Plates were incubated at 25°C for at least 7 days. Population densities were determined by counting the colony-forming units (CFU), and the results are reported as CFU per gram of fresh weight. Approximately 80 isolates per sample were randomly selected from R2A, purified by streak dilution and stored at −80°C in nutrient broth containing 40% glycerol until further processing.

### Dereplication and Phylogenetic Classification of Epiphytic and Endophytic Isolates

Matrix-assisted laser desorption/ionization time of flight mass spectrometry (MALDI-TOF MS) was used as an approved high-throughput method for the dereplication and classification of bacteria in large environmental studies (Huschek and Witzel, 2019). Prior to measurement, bacterial isolates were freshly inoculated on CASO broth (Fluka, Buchs, Switzerland) solidified with 15 g L^–1^ agar and cultivated for 24 h at 25°C. The whole-cell measurement protocol was used to obtain mass spectra. Briefly, ∼0.1 mg of cell material was transferred directly from the edge of a bacterial colony to a MALDI target spot. After drying at room temperature, sample spots were overlaid with 1 μl of matrix solution (Bruker Daltonics, Bremen, Germany). The MS analysis was performed with a Microflex^TM^ LT/SH MALDI-TOF mass spectrometer (Bruker Daltonics) using Flex Control 3.4 software, as described in detail by Müller et al. (2016). The MALDI Biotyper Preprocessing Standard Method and the MALDI Biotyper MSP Identification Standard Method were used for bacterial classification with Biotyper 3.1 software equipped with the MPS Library (release August 2018), according to the manufacturer’s instructions (Bruker Daltonics). Isolates with a score value greater than 2.3 (highly probable species identification) were considered as identified by the Bruker database. The spectra of the isolates with lower scores were compared with each other and grouped based on a score > 2.3. Representative strains of the unidentified groups were taxonomically classified by sequencing almost the complete 16S rRNA gene. Briefly, total DNA was extracted from single colonies by resuspending them in 20 μl of 25 mM NaOH/0.25% SDS and incubating the mixture for 15 min at 95°C. The 16S rRNA gene was amplified using primers 8f and 1525r (Lane, 1991) according a protocol described by Ulrich and Wirth (1999) and sequenced with the internal primers 1492r (Lane, 1991) and 782r (Chun and Goodfellow, 1995) or 926r (Liu et al., 1997). All reference strains were unambiguously taxonomically assigned at the genus level with mothur using the RDP database. For a further phylogenetic classification, the closest related species were determined using EzBioCloud (Yoon et al., 2017). After supplementing the MALDI Biotyper^TM^ database with the spectra of the reference strains, a reliable taxonomic identification of all isolates was achieved using the results of the MALDI-TOF MS analysis. The MALDI groups were designated with respect to the assigned reference strain. If several groups of the same bacterial species were identified, the groups were consecutively numbered. For groups assigned to one of our reference isolates, the species name is reported in quotation marks and represents the most closely related species (Supplementary Table S1).

The *Pseudomonas* MALDI groups were subjected to a further phylogenetic analysis using a multi-locus sequence analysis (MLSA). The housekeeping genes *gyrB*, *rpoB*, and *rpoD* were chosen for this purpose and amplified and sequenced as described by Mulet et al. (2010). Together with the 16S rRNA gene, a concatenated tree was constructed. The alignment comprised 1,432 bp of 16S rRNA, 1,088 bp of *gyrB*, 1,111 bp of *rpoB*, and 716 bp of *rpoD* gene. The total length of the alignment was 4,347 bp.

For the statistical analysis, the number of isolates per sample was normalized using the CSS method (Paulson et al., 2013). Significant differences in the community composition between tolerant and susceptible trees were tested by the MetagenomeSeq tool (MicrobiomeAnalyst, *P*_FDR_ < 0.05).

The 16S rRNA gene sequences of the reference isolates of the MALDI groups were deposited in NCBI (RRID: SCR_006472) under the accession numbers MN989032–MN989182.

### *In vitro* Screening and Statistical Analysis of Antagonistic Activity Toward *H. fraxineus*

All bacterial isolates were screened for their antagonistic activity toward *H. fraxineus* isolate P3 using a cocultivation assay described by Pane and Zaccardelli (2015). Briefly, an agar plug (ø 5 mm) with *H. fraxineus* mycelium was placed in the center of the petri dish, while four bacterial isolates were spread around the edge of the plate. Plates containing the *H. fraxineus* mycelium alone were used as control. The test was performed on PDA medium enriched with ash shoot extract (30 g of ash leaves per liter of PDA) at 22°C. Due to its slow growth, *H. fraxineus* was inoculated 5–6 days before the bacteria. The antifungal activity was monitored by measuring the fungal colony radius (*r*) after 1 and 2 weeks until the control reached the edge of the plate. The inhibition of *H. fraxineus* was estimated using the following formula: percent growth inhibition = 100 × [(*r*_control_ − *r*_cocultivation_) ÷ *r*_control_]. Isolates with inhibition rates >30% were chosen for a subsequent statistical assay (three replicates) with two *H. fraxineus* isolates (P3 and HF23). In this approach, each bacterial isolate was tested on a separate plate by spreading it around the fungus four times. The statistical test was also used to evaluate the vitality of the residual *H. fraxineus* mycelium after confrontation with the bacteria. For this purpose, agar plugs (ø 5 mm) of the mycelium were picked and incubated on PDA with the ash shoot extract. The growth of the fungus was assessed after 2 weeks by estimating the mycelium diameter and comparing it to the untreated control.

Two-year-old ash seedlings were inoculated with either isolate using an approach described by Przybyl (2003) to verify the pathogenicity of the *H. fraxineus* isolates. The symptoms were monitored for 8 weeks, and the virulence of both isolates could be confirmed.

## Results

### Comparison of the Bacterial Community Structure by Amplicon Sequencing of the 16S rRNA Gene

The V5–V6 region of the 16S rRNA gene from the microbiomes of 16 tolerant and 16 susceptible trees was amplified with primers that exclude the host plant DNA. An evaluation of the Illumina sequence reads revealed an exceedingly high abundance of the genera *Bacillus* (93%) or *Escherichia* and *Pseudomonas* (89%) in the samples B2K and A1K (from susceptible trees), respectively, suggesting that these samples were contaminated. Both samples were excluded from the analyses. After quality filtering and removing potential chimeras and plastid and mitochondrial sequences from ash trees, 3,562,425 high-quality sequences were obtained that formed 3,634 ASVs with an average of 413 ASVs per sample. Sequences rarefied to 44,537 reads per sample were used as input for all subsequent analyses.

**FIGURE 1:**
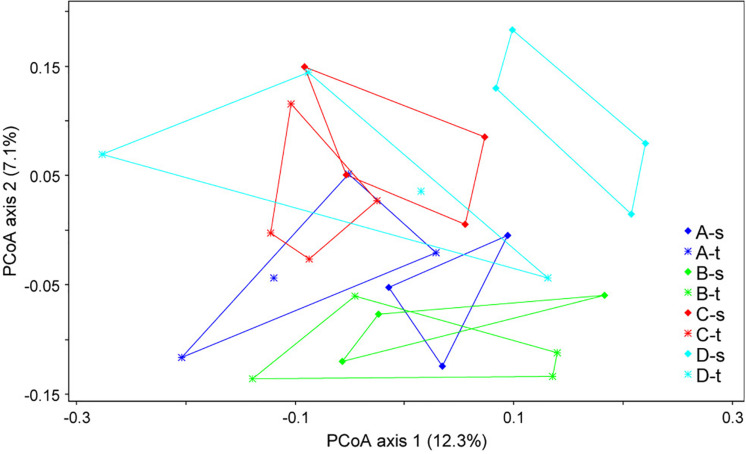
Ordination plot showing the differences in the community structures of tolerant (t) and susceptible (s) trees across the four sampled forest districts (A–D). A principal coordinate analysis (PCoA) was applied based on the unweighted UniFraq distance matrix.

Alpha diversity estimates such as the Shannon index showed no significant differences, with values of 4.02 ± 0.34 (mean ± SD) for susceptible trees and 4.03 ± 0.35 for tolerant trees. The analysis of the beta diversity resulted in small but significant differences between the bacterial community structure grouped by forest plot and health status (ANOSIM *R* = 0.2118, *P* < 0.001). The differences are shown in Figure 1. The first axis indicated some differences between tolerant and susceptible trees, particularly in plot D. However, the pairwise comparisons did not result in any significant differences.

The taxonomic assignment of ASVs revealed 10 taxa at the phylum level. The phylum Proteobacteria predominated across all samples (with a mean of 58%). Within the Proteobacteria, α-Proteobacteria were the most abundant class, representing 40% of the total sequences, followed by γ- and β-Proteobacteria at 9 and 6%, respectively. Other abundant phyla were Bacteroidetes (20%), Actinobacteria (17%), and Deinococcus–Thermus (3%). Firmicutes accounted for only 0.33% of ASVs among all samples. Only 1.8% of the total sequences were unable to be assigned to bacterial taxa. The results revealed a high phylogenetic diversity of leaf bacteria, with 241 taxa identified at the genus level. *Sphingomonas* (18.4%), *Hymenobacter* (16%), and bacteria of the order Rhizobiales (8.7%) dominated the total bacterial community. Other abundant genera were *Methylobacterium* (5.4%), *Pseudomonas* (3.2%), *Deinococcus* (3.0%), and *Massilia* (2.7%).

Comparing the bacterial microbiomes of tolerant and susceptible trees at the phylum level, Firmicutes was significantly (threefold) increased in tolerant *F. excelsior* (*P*_FDR_ = 0.05). At the class level, γ-Proteobacteria were present at significantly higher levels in tolerant plants (*P*_FDR_ = 0.046) and were particularly enriched with sequences associated with bacteria of the family Xanthomonadales (*P*_FDR_ = 0.0016) and the genus *Luteimonas* (*P*_FDR_ = 3.9E-06). Five abundant phylotypes (>0.3%) were significantly increased in tolerant trees at the genus level. *Luteimonas* showed a 15-fold increase, Halomonadaceae sp. a 3.8-fold increase, *Microbacterium* a 2.6-fold increase, *Aliihoeflea* a 27-fold increase, and *Agrococcus* a 2.2-fold increase. Some other genera, such as *Aureimonas*, were also present at higher levels (3.7-fold) in tolerant trees, but the difference was not significant.

**FIGURE 2:**
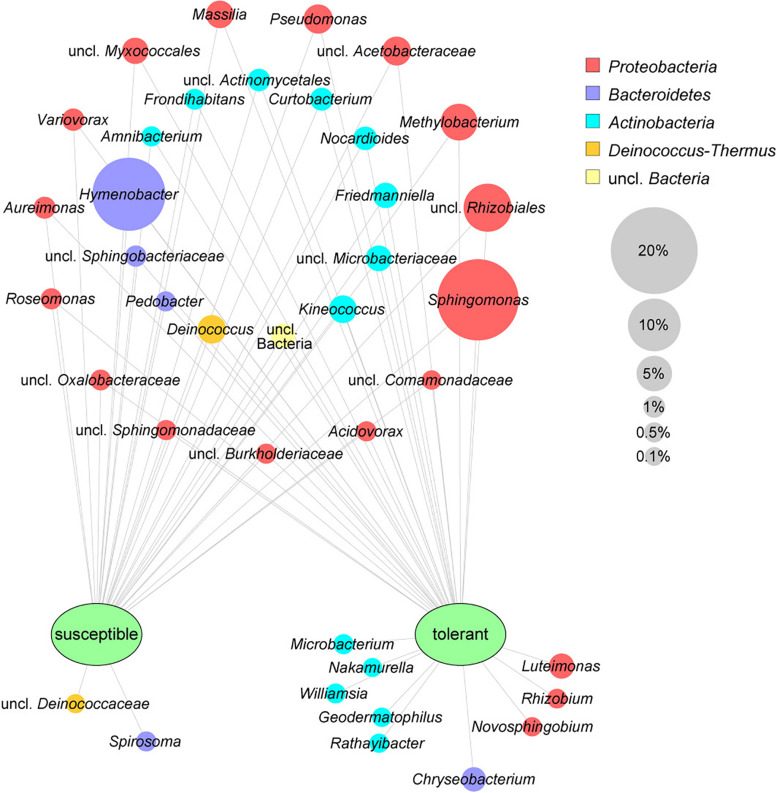
Core microbiomes of susceptible and tolerant *F. excelsior* leaves at the genus level. The core microbiomes (taxa occurring in 75% of all replicates of each group that exhibited at least 0.1% abundance in the community) were combined for the network analysis. The size of nodes corresponds to the relative abundance in the whole dataset, node labels display their taxonomic affiliation, and the color of the nodes indicates the respective phylum.

The leaf core microbiome was determined at the genus level and defined as those taxa that were present in at least 75% of the respective samples. It included 37 abundant bacterial taxa (greater than 0.1%) for the tolerant *F. excelsior* trees, which represent 87.1% of all sequences. Susceptible plants contained only 30 genera in their core microbiome (88.2% of the total sequences). The comparison of the core microbiomes of tolerant and susceptible ash trees is illustrated using a clustering network (Figure 2). Twenty-eight taxa shared both core microbiomes, including the predominant genera *Sphingomonas* and *Hymenobacter*. Nine genera from three different phyla were specific to the tolerant trees, with the highest abundance observed for *Luteimonas* and *Chryseobacterium*. Two genera were detected only in susceptible trees. Overall, the specific bacteria for trees with each health status were less abundant than the shared microbiota.

**FIGURE 3:**
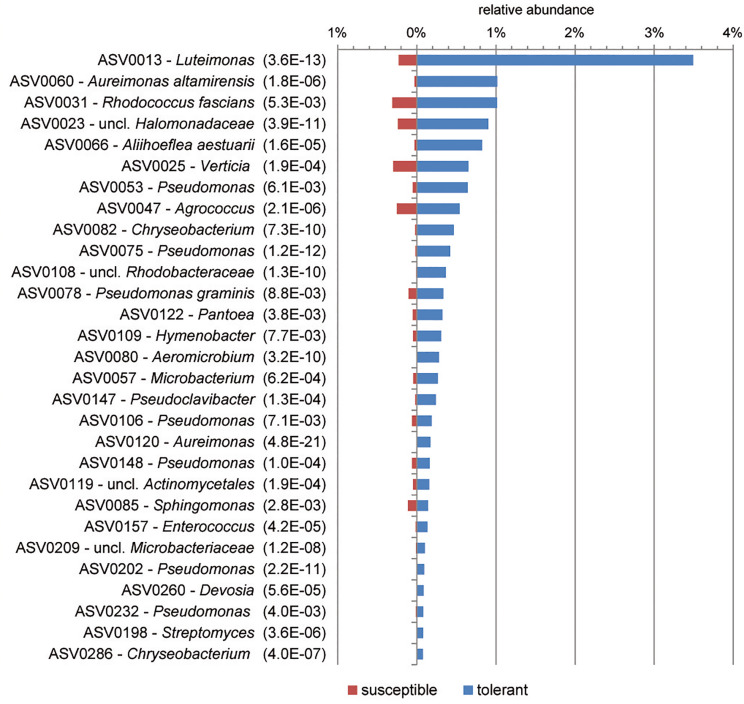
Overview of significantly increased amplicon sequence variants (ASVs) in tolerant *F. excelsior* trees. The ASVs are arranged according their abundance (>0.07%). Statistical significance (*P*_FDR_) is indicated in brackets.

Based on ASVs, bacterial communities of tolerant and susceptible trees were compared using a differential abundance analysis. Figure 3 presents an overview of ASVs that were significantly increased in tolerant ash trees. ASV0013, which was identified as *Luteimonas* sp., was remarkably increased in tolerant trees. Several ASVs from other genera were increased; for instance, seven ASVs of the genus *Pseudomonas* and two ASVs of *Aureimonas* showed a significantly higher abundance in tolerant trees.

### Comparison of the Culturable Bacterial Communities of Susceptible and Tolerant *F. excelsior* Trees

Population densities of culturable bacteria in *F. excelsior* leaves and petioles ranged from 5 × 10^4^ to 8 × 10^5^ CFU g^–1^ fresh weight. Significant differences were not observed between tolerant and susceptible trees.

The 2,589 bacterial isolates derived from the 32 samples represent the common culturable epiphytic and endophytic bacterial communities from the leaves of susceptible and tolerant *F. excelsior* trees. The isolates were classified into 166 phylotypes (MALDI groups) belonging to 45 genera (Supplementary Table S1). At the phylum level, the culturable ash-associated bacteria were dominated by Proteobacteria (81%), followed by Actinobacteria (14%) and Firmicutes (5%). Proteobacteria were mainly represented by α- and γ-Proteobacteria at 47% and 33%, respectively. In the comparison of tolerant and susceptible trees, the amount of Proteobacteria was the same, whereas the proportions of Actinobacteria (11% to 18%) and Firmicutes (9% to 1%) were different to some extent, as the latter was significantly increased in tolerant plants (*P*_FDR_ = 0.0044). Regarding the bacterial classes, γ-Proteobacteria (37 to 29%, *P*_FDR_ = 0.022) and Bacilli (9 to 1%, *P*_FDR_ = 0.021) were present at significantly higher levels in tolerant trees.

**FIGURE 4:**
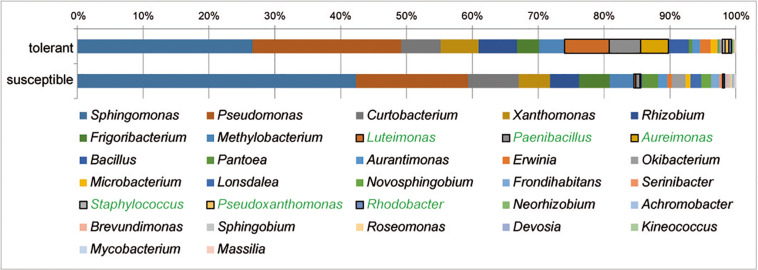
Structure of the culturable bacterial communities in tolerant and susceptible *F. excelsior* trees. Genera with significantly increased abundance in tolerant trees are highlighted in green. The analysis is based on the classification of 2,589 isolates (16 replicates).

At the genus level, *Sphingomonas* and *Pseudomonas* dominated the culturable bacterial community of ash trees at 33 and 20%, respectively, followed by *Curtobacterium*, *Xanthomonas*, and *Rhizobium* each with 6% and *Frigoribacterium* and *Luteimonas* with 4%. The relative abundance of bacterial genera in trees differing in health status is shown in Figure 4. A comparison of bacterial communities revealed a significantly higher proportion of isolates in tolerant trees for the abundant genera *Luteimonas* (24-fold), *Paenibacillus* (7-fold), and *Aureimonas* (46-fold), as well as genera occurring at lower percentages, such as *Staphylococcus*, *Pseudoxanthomonas*, and *Rhodobacter*.

**FIGURE 5:**
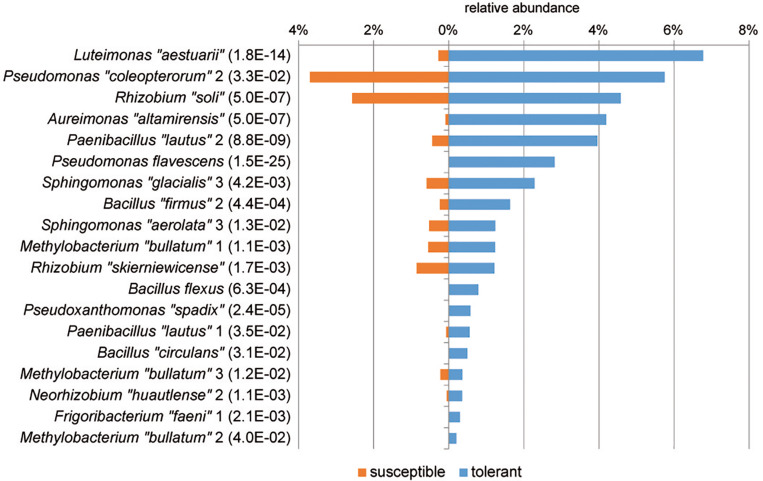
MALDI groups of bacterial isolates with a significantly higher abundance in tolerant trees. Groups displaying significant increases are sorted according to their abundance. The level of significance is indicated in brackets (*P*_FDR_, MetagenomeSeq). Species names enclosed in quotation marks were assigned based on 16S rRNA gene sequencing. Similarity values to the closest relative type strains are shown in Supplementary Table S1.

A differential abundance analysis was performed at the level of MALDI groups to identify groups of isolates specific for tolerant trees that may be associated with the ability of the tree to resist the pathogen (Figure 5). In tolerant trees, the *Luteimonas* MALDI group was increased more than 20-fold. Additionally, other groups, such as *Aureimonas* “*altamirensis*,” *Paenibacillus* “*lautus*” 2, *Pseudomonas flavescens*, *Sphingomonas* “*glacialis*” 3, and *Bacillus* “*firmus*” 2, showed a distinctly higher abundance in the culturable bacterial community of tolerant trees. Of the four *Methylobacterium* “*bullatum*” MALDI groups, three groups were significantly increased in tolerant trees.

**FIGURE 6:**
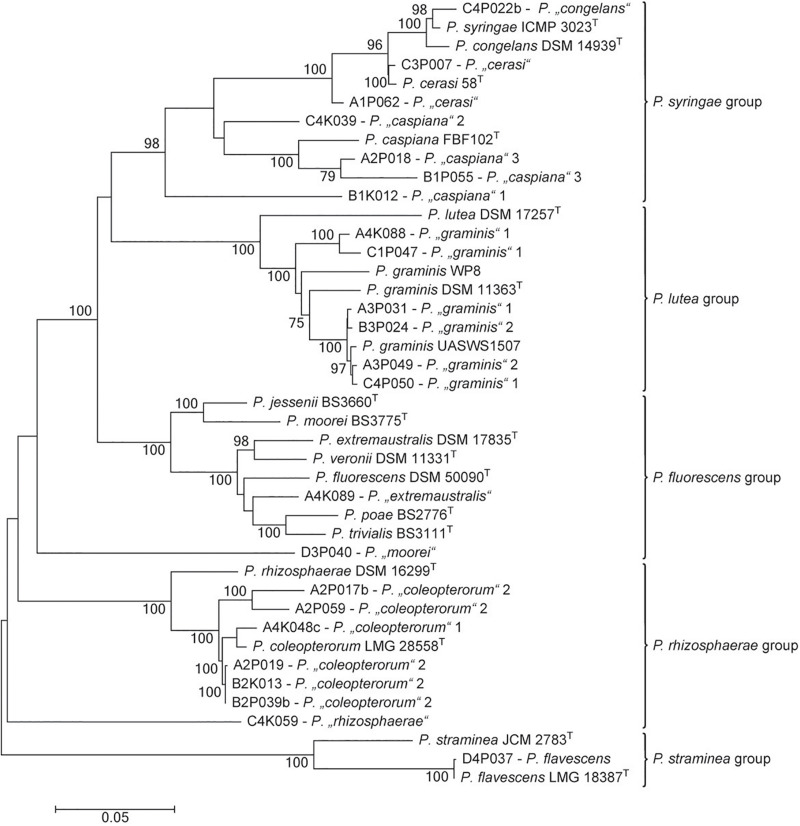
Phylogenetic tree of representative strains for the *Pseudomonas* MALDI groups in relation to respective type strains of the genus. The tree is based on the analysis of partial sequences of four concatenated genes (16S rRNA, *gyrB*, *rpoB*, and *rpoD*; accession numbers/locus tags are listed in Supplementary Table S2). The dendrogram was generated with the maximum likelihood algorithm based on the general time-reversible substitution model with G + I. Numbers at branch nodes refer to bootstrap values >70%. Bar, 0.01 substitutions per nucleotide site. The designation of MALDI groups was only based on 16S rRNA gene similarity. *Pseudomonas* species groups were indicated as proposed by Peix et al. (2018).

The genus *Pseudomonas* is particularly interesting among bacteria that are able to inhibit the activity of pathogenic fungi. It was the second-most abundant genus in the culturable bacterial community of ash leaves. In tolerant trees, the proportion of pseudomonads was slightly increased (22.6%) compared to that in susceptible trees (17%). Altogether, 497 *Pseudomonas* isolates were obtained from the 32 leaf samples and classified into 13 MALDI groups belonging to five different species groups (*Pseudomonas lutea*, *Pseudomonas rhizosphaerae*, *Pseudomonas syringae*, *Pseudomonas fluorescens*, and *Pseudomonas straminea*) within the *P. fluorescens* lineage (Mulet et al., 2010; Peix et al., 2018). For a more precise differentiation of the species groups, at least one representative isolate was chosen from each MALDI group to perform a MLSA (Figure 6). The analysis focused on *Pseudomonas coleopterorum* and *Pseudomonas graminis* because they contained the largest numbers of isolates (179 and 114, respectively). Eight of the 13 MALDI groups were clearly assigned to five distinct *Pseudomonas* species. In addition to *P. flavescens* and *Pseudomonas cerasi*, two MALDI groups were each classified into *P. coleopterorum*, *P. graminis*, and *Pseudomonas caspiana*. In contrast to the phylogenetic analysis based on the 16S rRNA gene, the *Pseudomonas* “*congelans*” group (isolate C4P022b) was located close to the *P. syringae*-type strain in the concatenated tree. Similarly, the *Pseudomonas* “*extremaustralis*” group (isolate A4K089) was more closely assigned to *Pseudomonas poae* and *Pseudomonas trivialis* than to the *P. extremaustralis*-type strain using MLSA. Three MALDI groups [*Pseudomonas* “*moorei*” (D4P040), *P.* “*rhizosphaerae*” (C4K059), and *P.* “*caspiana*” 1 (B1K012)] did not clearly cluster with any *Pseudomonas* strain, suggesting that these groups probably represent new species. Overall, the MALDI classification and MLSA result in a similar differentiation at the species level. Within a species, namely, *P. caspiana*, the differentiation corresponds to the MALDI groups. For *P. graminis* and *P. coleopterorum*, the intraspecies differentiation was not consistent between the two methods.

### Antagonistic Potential of Bacterial Isolates – Growth Inhibition of *H. fraxineus*

The bacterial isolates were screened for their ability to inhibit the growth of *H. fraxineus* using cocultivation assays as a method to detect putative antagonists. The screen produced 282 isolates (10.9% of the total isolates) that suppressed pathogen growth by at least 30%. In some cases, the inhibitory effect was combined with the lysis of the mycelium. The antagonistic isolates were obtained in the same proportions from tolerant and susceptible trees and mainly belonged to the genera *Sphingomonas* (38%) and *Pseudomonas* (21%). A series of other genera (18) from three different phyla, such as *Xanthomonas*, *Microbacterium*, and *Paenibacillus*, was present at comparably low proportions (1–7%, Supplementary Figure S1). Isolates of the genera *Pantoea* and *Bacillus* showed the highest mean growth inhibition rates (∼40%).

**FIGURE 7:**
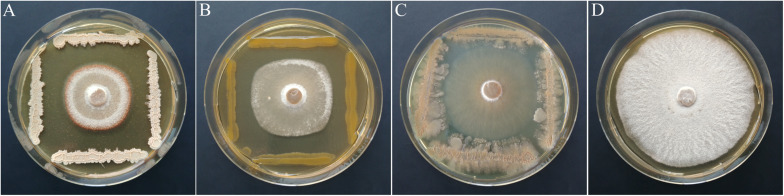
Inhibition of *H. fraxineus* P3 growth by cocultivation with *B.* “*velezensis*” A4P130 **(A)**, *P. vagans* B3K066 **(B)**, and *B. tequilensis* C4K066b **(C)**. The growth of *H. fraxineus* P3 without cocultivation served as the control **(D)**.

Based on the primary screen, 78 isolates were assayed for their antagonistic activity with a statistical cocultivation test using three replicates and two *H. fraxineus* strains. Both isolates were confirmed to be virulent on young ash trees, whereas *H. fraxineus* P3 was more susceptible in cocultivation tests than HF23 (Supplementary Table S3). The evaluation was completed by testing the vitality of the remaining fungal mycelium after confrontation with the bacteria. An overview of the activity of isolates from all tested MALDI groups is shown in Table 1. In general, the test confirmed the results of the screen, although several isolates, including members of the genus *Luteimonas*, failed in the statistical test. Of the 78 isolates, six strains significantly inhibited the growth of both *H. fraxineus* isolates, whereas 17 strains only affected P3 and eight only inhibited HF23 (Supplementary Table S3). The strongest effects were observed for *Bacillus* “*velezensis*” A4P130, *Pantoea* “*vagans*” B3K066, and *P.* “*caspiana*” B1P055, with growth inhibition rates ranging from 41 to 55%. In particular, cocultivation with A4P130 resulted in the formation of a sharp necrotic zone along the edge of the mycelium of *H. fraxineus* P3 (Figure 7). However, the mycelium picked outside of this zone remained viable. The isolates B3K066 and B1P055 exerted a dramatic effect by lysing the fungal mycelium during cocultivation. Accordingly, isolate P3 was completely killed, and the less susceptible isolate HF23 showed a visible reduction in vitality (Table 1). A clear antagonistic activity was also observed for *Bacillus* “*tequilensis*” C4K066b and *Erwinia* “*billingiae*” D4P109. These bacteria were weaker in their ability to immediately suppress fungal growth, but they affected the mycelium during cocultivation. For example, the mycelium of *H. fraxineus* P3 was completely lysed at the end of the confrontation with *B.* “*tequilensis*” C4K066b (Figure 7). This lytic effect was less pronounced for HF23, resulting in a relative high mycelium vitality (64%). In contrast, *E.* “*billingiae*” D4P109 completely killed both *H. fraxineus* isolates during cocultivation.

**TABLE 1:** Antagonistic activity of isolates from different MALDI groups assessed by the ability to inhibit the growth of *H. fraxineus* and vitality test of the remaining fungal mycelium.

Isolate^a^	Taxonomic assignment	Growth inhibition rate in coculture (%)^b^		Vitality of the mycelium (% of the untreated control)	
		P3	HF23	P3	HF23
C4K093	*Achromobacter* “*denitrificans*”	40.0*	12.3	98.4	81.5
A4P130	*Bacillus* “*velezensis*”	50.6*	54.7*	96.3	101.7
C4K066b	*Bacillus* “*tequilensis*”	35.1	37.5	0.0	63.9
C4P040b	*Bacillus cereus*	30.0	35.0	98.5	91.7
A2K052	*Curtobacterium* “*flaccumfaciens*” 2	28.4	22.9	0.0	87.5
D4P109	*Erwinia* “*billingiae*”	40.0*	22.5	0.0^c^	0.0^c^
B3K063	*Frigoribacterium* “*faeni*” 5	44.4*	37.5	0.0	82.9
D1P082	*Luteimonas* “*aestuarii*”	22.5	25.6	64.8	86.2
D3P076	*Methylobacterium* “*bullatum*” 1	30.0	2.4	100.0	90.7
C4K020	*Methylobacterium* “*cerastii*”	25.3	4.4	0.0^c^	94.4
D3P082	*Methylobacterium* “*goesingense*” 2	30.8	6.1	96.3	90.7
C4K087b	*Methylobacterium* “*pseudosasicola*” 2	22.1	18.8	97.1	90.3
C1P060	*Microbacterium* “*hatanonis*” 2	39.5*	17.7	95.8	96.0
D2K023	*Novosphingobium* “*fluoreni*” 4	35.0	34.6	85.9^c^	89.8
B3P038	*Paenibacillus* “*lautus*” 2	45.7*	7.3	67.6^c^	85.5
B3K066	*Pantoea* “*vagans*”	40.5*	47.5*	0.0	80.8
B1P055	*Pseudomonas* “*caspiana*” 3	41.0*	43.0*	0.0	86.8
A1P062	*Pseudomonas* “*cerasi*”	32.1	36.7	0.0	80.5
B2K013	*Pseudomonas* “*coleopterorum*” 2	32.1	26.8	0.0	1.9
D4P037	*Pseudomonas flavescens*	6.7	6.3	27.4^c^	92.9
A3P049	*Pseudomonas* “*graminis*” 1	29.6	17.5	0.0^c^	87.2
A4K089	*Pseudomonas* “*extremaustralis*”	31.3	21.5	0.0^c^	92.0
A2P026	*Pseudoxanthomonas* “*spadix*”	35.0	15.7	91.5	84.4
A2P086	*Rahnella* “*victoriana*”	33.3	26.2	0.0	97.4
B3P075	*Rhizobium* “*skierniewicense*”	31.3	30.0	0.0	86.7
B3P008	*Sphingomonas* “*aerolata*” 1	36.4	32.9	0.0^c^	100.0
D4P108	*Sphingomonas* “*taxi*” 1	22.5	35.0	98.6	100.0
D2K022	*Sphingomonas* “*aurantiaca*”	37.5	30.9	76.6^c^	95.2
B3K005	*Sphingomonas faeni* 1	38.7*	32.9	0.0	90.3
B4K076	*Variovorax* “*robiniae*” 1	26.7	26.8	96.0	88.7
A4P033	*Xanthomonas* “*cynarae*”	44.4*	28.9	0.0	81.0

^*a*^Of the 78 tested bacteria, only one typical or the best isolate from each MALDI group is listed. Details are provided in Supplementary Table S2. ^*b*^Growth inhibition was tested using the *H. fraxineus* isolates P3 and HF23. *Significantly increased compared to a control *Pseudomonas* sp. PI01 (culture collection Thünen Institute, Waldsieversdorf, Germany) that did not show antagonistic activity in cocultures (growth inhibition rates: 4.0 and 8.5). The test was performed using the Kruskal–Wallis one-way analysis of variance on ranks followed by pairwise comparisons with Dunn’s test (*P* < 0.05). ^*c*^Detection of the antagonistic isolate inside the recovered *H. fraxineus* mycelium or instead of the mycelium.

## Discussion

In ash dieback, which is caused by *H. fraxineus*, leaflets and petioles are the main entry point for the pathogen (Cleary et al., 2013; Hanackova et al., 2017b). During and directly after invasion and penetration of epidermal tissue, that is, during the biotrophic phase of the pathogen, endophytic and epiphytic microorganisms in both the leaf and the petiole might be able to inhibit the progression of the pathogen (Cross et al., 2017; Hanackova et al., 2017b; Mansfield et al., 2018). Therefore, microorganisms colonizing the compound leaves have the potential to function as biocontrol agents against *H. fraxineus*. Numerous studies have examined the association of fungi with *H. fraxineus* infections. However, studies of bacteria colonizing *F. excelsior* are rather rare, although bacteria are estimated to be the most abundant and diverse colonists of the leaf phyllosphere. In our study, population densities of up to 8 × 10^5^ CFU g^–1^ leaf were observed, which are in the same range as the values measured by Weyens et al. (2009). We analyzed endophytic and epiphytic bacteria using a combined approach based on the assumption that both groups may interact with the pathogen in a similar manner. In the phyllosphere, microbes are often observed both as epiphytes on the plant surface and as endophytes within plant tissue (Whipps et al., 2008; Hardoim et al., 2015; van Overbeek and Saikkonen, 2016). Many bacteria have the ability to switch between endophytic and free-living lifestyles and may help protect the plant from inside the leaves and from the leaf surface (Hardoim et al., 2008). Thus, in this context, the distinction between the two habitats appears arbitrary.

Bacterial communities in the ash phyllosphere were dominated by Proteobacteria, Bacteroidetes, and Actinobacteria, which have also been reported to be the dominant phyla in the phyllosphere of *F. excelsior* plants in the United Kingdom (Griffiths et al., 2020), as well as in other plants (Muller et al., 2016). In studies exclusively analyzing epiphytic bacteria in the phyllosphere of a number of trees, α-Proteobacteria dominated the bacterial community, consistent with our results (Redford et al., 2010; Laforest-Lapointe et al., 2016). Likewise, the proportion of Firmicutes was very low. An analysis of exclusively endophytic bacteria revealed a higher abundance of γ-Proteobacteria and Firmicutes (Hardoim et al., 2015).

At the genus level, similar to other woody plants such as poplar and grapevine, the microbiome of *F. excelsior* leaves was dominated by *Sphingomonas* and *Hymenobacter* (Leveau and Tech, 2011; Crombie et al., 2018). Aydogan et al. (2018) postulated that these genera may represent “hub” taxa that are very important for the microbiome structure and plant–pathogen interactions. In addition to *Sphingomonas*, isolates of the genus *Pseudomonas* dominated the culturable bacterial community in our study, while their proportions were very small among the ASVs. This finding indicates the importance of using classical cultivation methods to complement high-throughput sequencing approaches in studies examining the interaction between pathogens and the plant microbiota.

The main question to be answered in this study was whether differences exist in the microbiota of tolerant and susceptible trees that may be related to the severity of ash dieback. In the last few years, research examining the correlations between microorganisms and plant health has increased in importance and shifted from single strains and species to a more community-based view (Witzell and Martin, 2018; Schlechter et al., 2019). The identification and comparison of the core microbiomes of tolerant and susceptible trees are considered particularly important to detect key members of the microbial community involved in protecting the plants from pathogens and to estimate the spread of diseases in relation to microbiome interactions (Koskella et al., 2017; Lemanceau et al., 2017; Hamonts et al., 2018; Orozco-Mosqueda et al., 2018). Koskella et al. (2017) identified a link between the community composition of bark-associated bacteria and bleeding canker symptoms caused by *P. syringae* in horse chestnut, suggesting that tree microbiota are important to regulate the spread of the disease. In whitebark pine, the resistance to the fungal pathogen *Cronartium ribicola* is associated with microbiome combinations in healthy trees (Bullington et al., 2018). Similarly, interactions between *Erysiphe alphitoides*, the causal agent of oak powdery mildew, and foliar fungal and bacterial communities have been described for pedunculate oak (Jakuschkin et al., 2016). In the context of ash dieback, no significant differences were observed between the structures of the endophytic fungal communities of trees with and without symptoms using amplicon sequencing and culture-dependent methods (Hanackova et al., 2017a; Schlegel et al., 2018; Kosawang et al., 2019; Griffiths et al., 2020). Consistent with the study by Griffiths et al. (2020), the bacterial microbiome analyzed in our study did also not show a direct association between the health status and the community structure, but a number of bacterial groups were significantly associated with the *H. fraxineus* infection.

In the comparison of bacteria of tolerant and susceptible ash leaves at the phylum level, Firmicutes was significantly increased in tolerant leaves. Genera of this phylum are well-known potent antagonists and agents functioning in the biocontrol of pathogenic fungi by producing various bioactive metabolites, such as iturins and fengycins, with a strong inhibitory effect on the growth of a wide range of plant pathogens (Emmert and Handelsman, 1999; Ongena et al., 2005; Müller et al., 2015). In particular, isolates of *Bacillus* spp. are considered the most common groups that induce systemic resistance (ISR) (Vanpeer et al., 1991; Kloepper et al., 2004; Ongena et al., 2007) and prime plant defenses against pathogens (Elsayed et al., 2020). At the class level, γ-Proteobacteria showed a significantly higher abundance in tolerant *F. excelsior* trees. Consistent with this result, other studies have described γ-Proteobacteria as components of the microbiome of healthy oak and banana plants (Sapp et al., 2016; Köberl et al., 2017).

The most obvious differences at the genus or species level were observed for the γ-Proteobacterium *Luteimonas*, which was significantly increased in healthy trees using both amplicon sequencing (ASV 15-fold) and cultivation (MALDI group 24-fold). Isolates of this genus have been described as phyllosphere bacteria of different plants (Sun et al., 2012; Comby et al., 2017; Wemheuer et al., 2017). In antagonistic assays designed to identify biological control agents for the management of *Fusarium* head blight, *Luteimonas* showed a slight inhibition of pathogen growth (Comby et al., 2017). In our study, the genus *Luteimonas* was a component of the specific core microbiome of tolerant *F. excelsior* plants (Figure 2). Consistent with this finding, Köberl et al. (2017) described *Luteimonas* as part of the “healthy rhizosphere core microbiome” of banana plants in *Fusarium oxysporum* wilt-infested areas in Central America and as an indicator species of healthy plants.

Similar to *Luteimonas*, the abundance of *Aureimonas* was substantially increased in the leaves of tolerant plants (46-fold in the culturable microbiome and 3.7-fold in ASVs), suggesting that this genus might also be involved in the process that protects ash plants from the pathogen. *Aureimonas* strains were reported to possess antifungal activity against *Phytophthora nicotianae* in pineapple by González et al. (2017). Some species of this genus have been reported to produce siderophores (Lin et al., 2013), providing the plant with the ability to inhibit the growth of soilborne pathogens due to limited iron levels (Chaiharn et al., 2009), and these proteins also appear to be involved in activation of the ISR against foliar pathogens (van Loon et al., 2008).

Significantly higher amounts of isolates from the genus *Paenibacillus* were detected in tolerant trees. More specifically, two MALDI groups of *P.* “*lautus*” were distinctly increased. Various *Paenibacillus* species produce antifungal compounds such as iturins and paenimyxin (Chung et al., 2000; Selim et al., 2005) and excrete a variety of cell wall-degrading enzymes (Naing et al., 2014; Yadav and Dubey, 2018). *Paenibacillus* species are also known to elicit the ISR against pathogenic fungi (Sang et al., 2014; Grady et al., 2016).

In addition to these genera that are probably involved in the plant–pathogen interaction, the analysis revealed MALDI groups or ASVs of other genera, such as *Pseudomonas*, *Bacillus*, *Methylobacterium*, and *Sphingomonas*, present at a significantly higher abundance in the phyllosphere of tolerant *F. excelsior* leaves. In particular, *Pseudomonas* is noticeable because seven ASVs and two MALDI groups were significantly increased in the phyllosphere of tolerant plants. Bacteria of this genus are known to play an important role in the biological control of phytopathogenic fungi due to the production of antifungal metabolites and hydrogen cyanide (Bloemberg and Lugtenberg, 2001; Haas and Defago, 2005; Fouzia et al., 2015). Some species control the growth of pathogens by inhibiting spore germination (Raaijmakers et al., 1995) or by producing extracellular enzymes that lyse components of the fungal cell wall (Compant et al., 2005). In contrast to direct antagonism, different *Pseudomonas* strains have the capacity to inhibit or suppress the activity of pathogens by eliciting resistance pathways in the host plant (Bakker et al., 2007).

In the core microbiome, the Bacteroidetes member *Chryseobacterium* and several actinobacterial genera, such as *Microbacterium*, were also specific for tolerant ash trees (Figure 2). These genera are known not only for their abilities to promote plant growth but also as biocontrol agents targeting fungal pathogens (Borah et al., 2018; Sang et al., 2018).

Overall, the comparison of the microbiota of *F. excelsior* leaves revealed several bacterial groups that are specific for tolerant trees, such as *Luteimonas*, *Aureimonas*, *Pseudomonas*, *Bacillus*, and *Paenibacillus*, suggesting that some of these genera might be involved in the process that prevents the penetration and spread of the *H. fraxineus.*

The drivers for the differences between the microbiomes of tolerant and susceptible plants are not yet known. Besides stochastic colonization, long-living trees seem to be able to actively recruit useful microbial communities (Witzell and Martin, 2018). So the plant genotype could indirectly mediate the resistance toward the pathogen. However, other factors or a combination of both might be responsible for shaping the microbiota as well.

A screen against *H. fraxineus* was performed to identify antagonistic bacteria for use in subsequent *in planta* assays. One tenth of the total isolates suppressed the growth of *H. fraxineus* in the screen, but the antagonistic activity was confirmed in a statistical test for only a small proportion (approximately 1–2%) of the total isolates. Comparable studies of the antagonistic effects of bacteria toward *H. fraxineus* have not been published to date. Among the culturable fungal community, a large percentage of endophytes, particularly fast-growing isolates, inhibited the pathogen (32 or 75%), but, on the other hand, approximately 25% of the fungi were inhibited by *H. fraxineus* (Schulz et al., 2015; Hanackova et al., 2017a). The inhibition might be due to the production of different fungistatic compounds such as viridin and hymenosetin by the pathogen (Andersson et al., 2010; Halecker et al., 2014). Hymenosetin, a member of the family of 3-decalinoyltetramic acid antibiotics, also exhibited strong activity against gram-positive bacteria, whereas gram-negative bacteria were not affected (Halecker et al., 2014). However, in our cocultivation tests, the growth of the bacterial strains was not inhibited. Strikingly, the *H. fraxineus* strains P3 and HF23 used in this study displayed extremely different susceptibilities to the bacteria. This difference corresponds to the highly varying capabilities of *H. fraxineus* strains, including exoenzyme profiles, growth rates, and the production of antibiotics (Halecker et al., 2014; Junker et al., 2017).

The strongest growth inhibition was observed for a *B. velezensis* isolate; however, the vitality of the *H. fraxineus* mycelium was not substantially altered. On the other hand, the strong fungistatic effect might be sufficient to prevent the spread of the pathogen in ash leaves. *B. velezensis* is known to possess antagonistic activity toward various phytopathogenic fungi by producing antifungal compounds, such as bacillomycin, fengycin, and iturin (Palazzini et al., 2016; Lim et al., 2017). In tomato, Elsayed et al. (2020) showed a shift in the composition of the prokaryotic community in response to inoculation with *B. velezensis* and assumed a priming effect of plant defenses against the pathogen *Ralstonia solanacearum*.

*In vitro* screens for antagonism with dual cultures are a promising method to search for potential biocontrol candidates that directly affect the respective fungal pathogen. However, niche competition, blocking potential entry points of the pathogen, and indirectly activating the plant immune system also lead to a higher tolerance of the host plant (van Loon et al., 2008; Berg et al., 2017; Terhonen et al., 2018). Thus, non-antagonistic bacteria are important in plant defenses as well (De Boer et al., 2006; Ardanov et al., 2012; Ab Rahman et al., 2018). In recent years, inoculation with individual bacterial isolates has been shown to trigger a shift in the plant microbiome (Andreote et al., 2010; Mitter et al., 2017). According to Ardanov et al. (2012), an endophytic *Methylobacterium* isolate shifts the structure of the entire endophytic community in potato plants. The alteration in the microbiome correlated with the resistance to different pathogens, whereas the bacterium itself did not possess antagonistic activity.

In conclusion, the study of the ash phyllosphere resulted in the identification of bacteria specific to healthy leaves and confirmed the hypothesis that significant differences exist in a series of bacterial groups putatively capable of suppressing disease progression. This result was completed by a set of isolates that substantially inhibited *H. fraxineus* growth *in vitro*. The bacteria were isolated directly from ash leaves, wherefore it can be assumed that they are able to stably establish after inoculation of *F. excelsior* seedlings. In a next step, *in planta* experiments over a longer period of exposure to *H. fraxineus* are required to evaluate the isolates or consortia of isolates, acting via direct antagonism or competition or indirectly via induction of resistance.

## Data Availability Statement

The datasets generated for this study can be found in the Sequence Read Archive (SRA): PRJNA602193, NCBI: MN989032–MN989182.

## Author Contributions

KU and RB performed the experiments, analyzed the data, and prepared the manuscript. UB performed the MALDI-TOF manuscript analysis. KU, MK, and AU conducted the microbiome analysis. All authors listed substantially contributed to and approved the manuscript. AU and MK supervised the entire study.

## Conflict of Interest

The authors declare that the research was conducted in the absence of any commercial or financial relationships that could be construed as a potential conflict of interest.
