# Supplementary material for: A Comparative Analysis of Ash Leaf-Colonizing Bacterial Communities Identifies Putative Antagonists of Hymenoscyphus fraxineus
Source: Front Microbiol. 2020 May 29;11:966. doi: 10.3389/fmicb.2020.00966 (PMC7273808; doi:10.3389/fmicb.2020.00966)
Supplement: Supplementary file 1 [file Data_Sheet_1.docx]

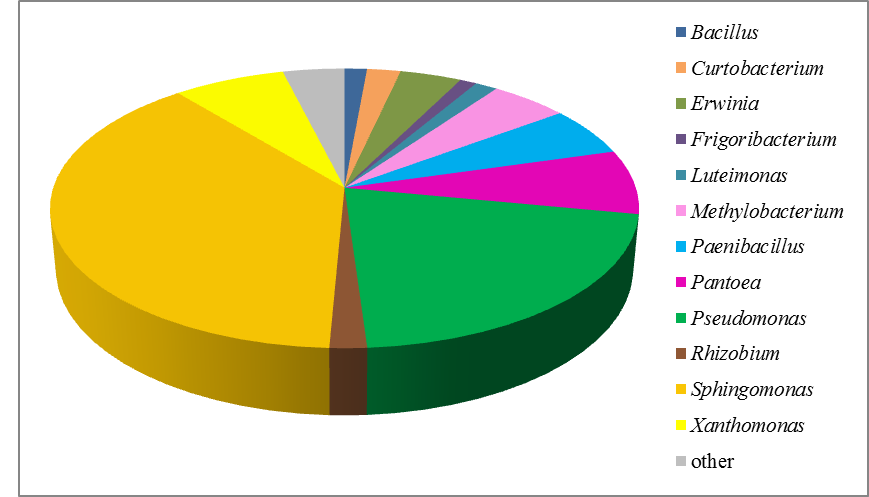


**Figure S1:** Relative abundance of bacterial genera among the antagonistic isolates. The data are the results of the primary screen and include isolates that inhibited the growth of *H. fraxineus* by greater than 30% (282 isolates). Other genera include *Achromobacter, Aurantimonas, Microbacterium, Neorhizobium, Novosphingobium, Pseudoxanthomonas, Rahnella* and *Variovorax.*

**Table S1:** Taxonomic affiliations of the identified MALDI groups.

| *MALDI group* | Reference isolate or strain | Best hit (EzBioCloud 16S database) | % | Highest similarity to a type strain | % |
| --- | --- | --- | --- | --- | --- |
| *Achromobacter "anxifer"* 1 | B3K054 | *Achromobacter* sp. B5 | 98.78 | *Achromobacter anxifer* LMG 26857 | 97.77 |
| *Achromobacter "anxifer"* 2 | C4P071b | *Achromobacter* sp. B5 | 98.64 | *Achromobacter anxifer* LMG 26857 | 97.77 |
| *Achromobacter "denitrificans"* | C3K027a | *Achromobacter denitrificans* DSM 30026^T^ | 97.91 | *Achromobacter denitrificans* DSM 30026 | 97.91 |
| *Aeromicrobium "ginsengisoli"* | B3K072 | *Aeromicrobium ginsengisoli* Gsoil 098^T^ | 99.50 | *Aeromicrobium ginsengisol*i Gsoil 098 | 99.50 |
| *Agrococcus "jejuensis"* | C3P020 | *Agrococcus jejuensis* DSM 22002^T^ | 99.93 | A*grococcus jejuensis* DSM 22002 | 99.93 |
| *Amnibacterium "soli"* | D1K005 | *Amnibacterium soli* MB78 | 99.79 | *Amnibacterium kyonggiense* KSL51201-037 | 98.67 |
| *Aurantimonas "coralicida"* | A4P024 | *Aurantimonas coralicida* DSM 14790^T^ | 97.61 | *Aurantimonas coralicida* DSM 14790 | 97.61 |
| *Aureimonas "altamirensis"* | C2P036 | *Aureimonas altamirensis* DSM 21988^T^ | 99.36 | *Aureimonas altamirensis* DSM 21988 | 99.36 |
| *Aureimonas "glaciei"* 1 | B4K049a | *Aureimonas glaciei* B5-2^T^ | 98.12 | *Aureimonas glaciei* B5-2 | 98.12 |
| *Aureimonas "glaciei"* 2 | D1K028 | *Aureimonas glaciei* B5-2^T^ | 98.13 | *Aureimonas glaciei* B5-2 | 98.13 |
| *Aureimonas "glaciei"* 3 | C4P083 | *Aureimonas glaciei* B5-2^T^ | 98.79 | *Aureimonas glaciei* B5-2 | 98.79 |
| *Bacillus "badius"* | D1P012 | *Bacillus badius* MTCC 1458^T^ | 99.93 | *Bacillus badius* MTCC 1458 | 99.93 |
| *Bacillus "circulans"* | C4P019a | *Bacillus* sp. Mt7 | 100.00 | *Bacillus circulans* ATCC 4513 | 99.59 |
| *Bacillus "firmus"* 1 | B4P005 | *Bacillus firmus* NBRC 15306^T^ | 98.84 | *Bacillus firmus* NBRC 15306 | 98.84 |
| *Bacillus "firmus"* 2 | B4P095b | *Bacillus firmus* NBRC 15306^T^ | 99.79 | *Bacillus firmus* NBRC 15306 | 99.79 |
| *Bacillus "tequilensis"* | C4K066b | *Bacillus tequilensis* KCTC 13622^T^ | 100.00 | *Bacillus tequilensis* KCTC 13622 | 100.00 |
| *Bacillus "velezensis"* | A4P130 | *Bacillus velezensis* CR-502^T^ | 99.86 | *Bacillus velezensis* CR-502 | 99.86 |
| *Bacillus cereus* | *Bacillus cereus* CICC 23949 | - | - | - | - |
| *Bacillus flexus* | *Bacillus flexus* DSM 1320^T^ | - | - | - | - |
| *Bacillus licheniformis* | *Bacillus licheniformis* CS 54_1 | - | - | - | - |
| *Bacillus* sp. | *Bacillus* sp. CICC 23998 | - | - | - | - |
| *Bacillus subtilis* | *Bacillus subtilis* DSM 10^T^ | - | - | - | - |
| *Brevundimonas "diminuta"* | A3K048a | *Brevundimonas diminuta* ATCC 11568^T^ | 99.63 | *Brevundimonas diminuta* ATCC 11568 | 99.63 |
| *Brevundimonas "intermedia"* | A1P056 | *Brevundimonas intermedia* ATCC 15262^T^ | 99.71 | *Brevundimonas intermedia* ATCC 15262 | 99.71 |
| *Brevundimonas "staleyi"* | B3P097 | *Brevundimonas staleyi* FWC43^T^ | 98.40 | *Brevundimonas staleyi* FWC43 | 98.40 |
| *Chryseobacterium "gregarium"* | C4P091 | *Chryseobacterium gregarium* P 461/12^T^ | 99.79 | *Chryseobacterium gregarium* P 461/12 | 99.79 |
| *Curtobacterium "ammoniigenes"* | A2K005 | *Curtobacterium* sp. Leaf261 | 99.93 | *Curtobacterium ammoniigenes* NBRC 101786 | 98.87 |
| *Curtobacterium "flaccumfaciens"* 1 | A2K029 | *Curtobacterium flaccumfaciens* LMG 3645^T^ | 98.94 | *Curtobacterium flaccumfaciens* LMG 3645 | 98.94 |
| *Curtobacterium "flaccumfaciens"* 2 | A4K043 | *Curtobacterium flaccumfaciens* LMG 3645^T^ | 99.35 | *Curtobacterium flaccumfaciens* LMG 3645 | 99.35 |
| *Curtobacterium "flaccumfaciens"* 3 | B2P067 | *Curtobacterium flaccumfaciens* LMG 3645^T^ | 99.79 | *Curtobacterium flaccumfaciens* LMG 3645 | 99.79 |
| *Curtobacterium "herbarum"* | D4K064a | *Curtobacterium herbarum* P 420/07^T^ | 99.09 | *Curtobacterium herbarum* P 420/07 | 99.09 |
| *Devosia "glacialis"* | C1K043 | *Devosia* sp. 1566 | 98.84 | *Devosia glacialis* Cr4-44 | 98.48 |
| *Erwinia "billingiae"* | A3K048b | *Erwinia billingiae* CIP 106121^T^ | 100.00 | *Erwinia billingiae* CIP 106121 | 100.00 |
| *Frigoribacterium "faeni"* 1 | A1P073 | *Frigoribacterium faeni* NBRC 103066^T^ | 99.93 | *Frigoribacterium faeni* NBRC 103066 | 99.93 |
| *Frigoribacterium "faeni"* 2 | A2K018 | *Frigoribacterium faeni* NBRC 103066^T^ | 99.15 | *Frigoribacterium faen*i NBRC 103066 | 99.15 |
| *Frigoribacterium "faeni"* 3 | A1P094 | *Frigoribacterium faeni* NBRC 103066^T^ | 99.08 | *Frigoribacterium faeni* NBRC 103066 | 99.08 |
| *Frigoribacterium "faeni"* 4 | C3K030 | *Frigoribacterium faeni* NBRC 103066^T^ | 99.37 | *Frigoribacterium faeni* NBRC 103066 | 99.37 |
| *Frigoribacterium "faeni"* 5 | A4P028 | *Frigoribacterium faeni* NBRC 103066^T^ | 99.57 | *Frigoribacterium faeni* NBRC 103066 | 99.57 |
| *Frondihabitans "peucedani"* 1 | A4K031 | *Frondihabitans* sp. PhB161 | 99.79 | *Frondihabitans peucedani* RS-15 | 98.59 |
| *Frondihabitans "peucedani"* 2 | A4K040 | *Frondihabitans* sp. PhB188 | 100.00 | *Frondihabitans peucedani* RS-15 | 98.70 |
| *Frondihabitans "sucicola"* | D4K023 | *Frondihabitans* sp. 762G35 | 98.95 | *Frondihabitans sucicola* GRS42 | 98.53 |
| *Herbiconiux "flava"* | A3P073 | *Herbiconiux flava* NBRC 16403^T^ | 99.01 | *Herbiconiux flava* NBRC 16403 | 99.01 |
| *Kineococcus "gynurae"* | B3K003 | *Kineococcus gynurae* KKD096^T^ | 97.54 | *Kineococcus gynurae* KKD096 | 97.54 |
| *Kineococcus "radiotolerans"* | B3K081a | *Kineococcus radiotolerans* SRS30216^T^ | 99.22 | *Kineococcus radiotolerans* SRS30216 | 99.22 |
| *Leifsonia "shinshuensis"* | PB4K024 | *Leifsonia shinshuensis* JCM 10591^T^ | 100.00 | *Leifsonia shinshuensis* JCM 10591 | 100.00 |
| *Lonsdalea "britannica"* | C2K034 | *Lonsdalea britannica* LMG 26267^T^ | 99.93 | *Lonsdalea britannica* LMG 26267 | 99.93 |
| *Luteimonas "aestuarii"* | A1P058 | *Luteimonas* sp. NML93-0399 | 99.65 | *Luteimonas aestuarii* B9 | 98.58 |
| *Luteimonas "terrae"* | C3K095 | *Luteimonas* sp. NML93-0399 | 99.72 | *Luteimonas terrae* THG-MD21 | 98.61 |
| *Massilia "violaceinigra"* 1 | A2K053 | *Massilia violaceinigra* B2^T^ | 98.54 | *Massilia violaceinigra* B2 | 98.54 |
| *Massilia "violaceinigra"* 2 | B2P089 | *Massilia violaceinigra* B2^T^ | 98.39 | *Massilia violaceinigra* B2 | 98.39 |
| *Methylobacterium "bullatum"* 1 | A3K044 | *Methylobacterium bullatum* F3.2^T^ | 100.00 | *Methylobacterium bullatum* F3.2 | 100.00 |
| *Methylobacterium "bullatum"* 2 | B2P072 | *Methylobacterium bullatum* F3.2^T^ | 99.85 | *Methylobacterium bullatum* F3.2 | 99.85 |
| *Methylobacterium "bullatum"* 3 | A1P099 | *Methylobacterium bullatum* F3.2^T^ | 99.85 | *Methylobacterium bullatum* F3.2 | 99.85 |
| *Methylobacterium "bullatum"* 4 | B4P014 | *Methylobacterium bullatum* F3.2^T^ | 100.00 | *Methylobacterium bullatum* F3.2 | 100.00 |
| *Methylobacterium "cerastii"* | A1K060 | *Methylobacterium cerastii* C44^T^ | 98.59 | *Methylobacterium cerastii* C44 | 98.59 |
| *Methylobacterium "goesingense"* 1 | C1K016 | *Methylobacterium goesingense* iEII3^T^ | 98.42 | *Methylobacterium goesingense* iEII3 | 98.42 |
| *Methylobacterium "goesingense"* 2 | B1P093 | *Methylobacterium goesingense* iEII3^T^ | 99.63 | *Methylobacterium goesingense* iEII3 | 99.63 |
| *Methylobacterium "goesingense"* 3 | A1K038a | *Methylobacterium goesingense* iEII3^T^ | 99.57 | *Methylobacterium goesingense* iEII3 | 99.57 |
| *Methylobacterium "goesingense"* 4 | D3P083 | *Methylobacterium goesingense* iEII3^T^ | 99.71 | *Methylobacterium goesingense* iEII3 | 99.71 |
| *Methylobacterium "gossipiicola"* | B3K042 | *Methylobacterium gossipiicola* Gh-105^T^ | 99.13 | *Methylobacterium gossipiicola* Gh-105 | 99.13 |
| *Methylobacterium "marchantiae"* 1 | C1K002 | *Methylobacterium marchantiae* JT1^T^ | 99.14 | *Methylobacterium marchantiae* JT1 | 99.14 |
| *Methylobacterium "marchantiae"* 2 | A3K057 | *Methylobacterium marchantiae* JT1^T^ | 99.85 | *Methylobacterium marchantiae* JT1 | 99.85 |
| *Methylobacterium "pseudosasicola"* 1 | C1P061 | *Methylobacterium pseudosasicola* BL36^T^ | 99.28 | *Methylobacterium pseudosasicola* BL36 | 99.28 |
| *Methylobacterium "pseudosasicola"* 2 | D2P087 | *Methylobacterium pseudosasicola* BL36^T^ | 99.57 | *Methylobacterium pseudosasicola* BL36 | 99.57 |
| *Methylobacterium mesophilicum* | *Methylobacterium mesophilicum* MB162 | - | - | - | - |
| *Microbacterium "aoyamense"* | C3K055 | *Microbacterium aoyamense* KV-492^T^ | 99.72 | *Microbacterium aoyamense* KV-492 | 99.72 |
| *Microbacterium "hatanonis"* 1 | C4P033 | *Microbacterium hatanonis* JCM 14558^T^ | 100.00 | *Microbacterium hatanonis* JCM 14558 | 100.00 |
| *Microbacterium "hatanonis"* 2 | C3K049 | *Microbacterium hatanonis* JCM 14558^T^ | 100.00 | *Microbacterium hatanonis* JCM 14558 | 100.00 |
| *Moraxella "osloensis"* | C2P011 | *Moraxella osloensis* CCUG 350^T^ | 99.51 | *Moraxella osloensis* CCUG 350 | 99.51 |
| *Mycobacterium "canariasense"* | B4K041 | *Mycolicibacterium canariasense* JCM 15298^T^ | 99.28 | *Mycolicibacterium canariasense* JCM 15298 | 99.28 |
| *Nakamurella "flavida"* | A3P061 | *Nakamurella flavida* DS-52^T^ | 99.93 | *Nakamurella flavida* DS-52 | 99.93 |
| *Neorhizobium "alkalisoli"* | B4K019 | *Neorhizobium alkalisoli* CCBAU 01393^T^ | 98.85 | *Neorhizobium alkalisoli* CCBAU 01393 | 98.85 |
| *Neorhizobium "huautlense"* 1 | A1K065 | *Neorhizobium* sp. SPY-1 | 98.04 | *Neorhizobium huautlense* S02 | 97.83 |
| *Neorhizobium "huautlense"* 2 | A4P021 | *Neorhizobium* sp. SPY-1 | 98.05 | *Neorhizobium huautlense* S02 | 97.83 |
| *Novosphingobium "barchaimii"* | B4K030 | *Novosphingobium* *barchaimii* LL02^T^ | 99.21 | *Novosphingobium barchaimii* LL02 | 99.21 |
| *Novosphingobium "fluoreni"* 1 | B4K084a | *Novosphingobium* sp. BF0001B031^T^ | 99.29 | *Novosphingobium fluoreni* HLJ-RS18 | 97.93 |
| *Novosphingobium "fluoreni"* 2 | A1K012 | *Novosphingobium* sp. BF0001B031^T^ | 99.57 | *Novosphingobium fluoreni* HLJ-RS18 | 98.20 |
| *Novosphingobium "fluoreni"* 3 | B3P002 | *Novosphingobium* sp. BF0001B031^T^ | 99.50 | *Novosphingobium fluoreni* HLJ-RS18 | 98.13 |
| *Novosphingobium "fluoreni"* 4 | D2K023 | *Novosphingobium* sp. BF0001B031^T^ | 99.78 | *Novosphingobium fluoreni* HLJ-RS18 | 98.49 |
| *Okibacterium "fritillariae "* 1 | A3P100 | *Okibacterium* *fritillariae* VKM Ac-2059^T^ | 99.79 | *Okibacterium fritillariae* VKM Ac-2059 | 99.79 |
| *Okibacterium "fritillariae "* 2 | D4K016 | *Okibacterium* *fritillariae* VKM Ac-2059^T^ | 100.00 | *Okibacterium fritillariae* VKM Ac-2059 | 100.00 |
| *Paenibacillus "glucanolyticus"* | B1P042a | *Paenibacillus* *glucanolyticus* DSM 5162^T^ | 99.72 | *Paenibacillus glucanolyticus* DSM 5162 | 99.72 |
| *Paenibacillus "lautus"* 1 | C3K061 | *Paenibacillus* sp. Y412MC10 | 99.66 | *Paenibacillus lautus* NBRC 15380 | 99.45 |
| *Paenibacillus "lautus"* 2 | A4P128 | *Paenibacillus* *lautus* NBRC 15380^T^ | 99.58 | *Paenibacillus lautus* NBRC 15380 | 99.58 |
| *Paenibacillus "phoenicis"* | C3P090 | *Paenibacillus* *phoenicis* 3PO2SA^T^ | 99.86 | *Paenibacillus phoenicis* 3PO2SA | 99.86 |
| *Paenibacillus lactis* | *Paenibacillus lactis* DSM 15596^T^ | - | - | - | - |
| *Paenibacillus phoenicis* | *Paenibacillus phoenicis* NF_02 | - | - | - | - |
| *Pantoea "vagans"* | B3K079a | *Pantoea* *vagans* LMG 24199^T^ | 99.71 | *Pantoea vagans* LMG 24199 | 99.71 |
| *Plantibacter "flavus "* | A2K032 | *Plantibacter* *flavus* VKM Ac-2504^T^ | 100.00 | *Plantibacter flavus* VKM Ac-2504 | 100.00 |
| *Pseudomonas "caspiana"* 1 | B1K012 | *Pseudomonas* *caspiana* FBF102^T^ | 99.44 | *Pseudomonas caspiana* FBF102 | 99.44 |
| *Pseudomonas "caspiana"* 2 | C4K039 | *Pseudomonas* sp. Irchel 3A5 | 99.79 | *Pseudomonas caspiana* FBF102 | 99.65 |
| *Pseudomonas "caspiana"* 3 | B1P055 | *Pseudomonas* sp. Irchel 3A5 | 99.93 | *Pseudomonas caspiana* FBF102 | 99.79 |
| *Pseudomonas "cerasi"* | A1P062 | *Pseudomonas* *cerasi* 58^T^ | 99.72 | *Pseudomonas cerasi* 58 | 99.72 |
| *Pseudomonas "coleopterorum"* 1 | A4K048c | *Pseudomonas* *coleopterorum* Esc2Am^T^ | 99.52 | *Pseudomonas coleopterorum* Esc2Am | 99.52 |
| *Pseudomonas "coleopterorum"* 2 | B2P039b | *Pseudomonas* *coleopterorum* Esc2Am^T^ | 99.93 | *Pseudomonas coleopterorum* Esc2Am | 99.93 |
| *Pseudomonas "extremaustralis"* | A4K089 | *Pseudomonas* sp. 286 | 99.86 | *Pseudomonas extremaustralis* 14-3 | 99.79 |
| *Pseudomonas "congelans"* | C4P022b | *Pseudomonas congelans* DSM 14939^T^ | 99.93 | *Pseudomonas congelans* DSM 14939 | 99.93 |
| *Pseudomonas "graminis"* 1 | A3P029 | *Pseudomonas* sp. UASWS1507 | 100.00 | *Pseudomonas graminis* DSM 11363 | 99.86 |
| *Pseudomonas "graminis"* 2 | B3P024 | *Pseudomonas* *graminis* DSM 11363^T^ | 99.93 | *Pseudomonas graminis* DSM 11363 | 99.93 |
| *Pseudomonas "moorei"* | D3P040 | *Pseudomonas* sp. ASAD | 98.68 | *Pseudomonas moorei* RW10 | 98.67 |
| *Pseudomonas "rhizosphaerae"* | C4K059 | *Pseudomonas* sp. 286 | 99.03 | *Pseudomonas rhizosphaerae* DSM 16299 | 98.96 |
| *Pseudomonas flavescens* | *Pseudomonas flavescens* DSM 12071^T^ | - | - | - | - |
| *Pseudoxanthomonas "spadix"* | A2P100 | *Pseudoxanthomonas* *spadix* DSM 18855^T^ | 98.97 | *Pseudoxanthomonas spadix* DSM 18855 | 98.97 |
| *Rahnella "victoriana"* | A2P014 | *Rahnella* *victoriana* FRB 225^T^ | 99.78 | *Rahnella victoriana* FRB 225 | 99.78 |
| *Rhizobium "naphthalenivorans"* | A3K072 | *Rhizobium* *naphthalenivorans* TSY03b^T^ | 96.83 | *Rhizobium naphthalenivorans* TSY03b | 96.83 |
| *Rhizobium "qilianshanense"* | B4K095 | *Rhizobium* sp. Leaf371 | 99.72 | *Rhizobium qilianshanense* CCNWQLS01 | 97.28 |
| *Rhizobium "skierniewicense"* | B2P050 | *Rhizobium* *skierniewicense* Ch11^T^ | 99.71 | *Rhizobium* *skierniewicense* Ch11 | 99.71 |
| *Rhizobium "smilacinae"* | A4P062 | *Rhizobium* *smilacinae* PTYR-5^T^ | 99.78 | *Rhizobium smilacinae* PTYR-5 | 99.78 |
| *Rhizobium "soli"* | A2P052 | *Rhizobium* *soli* DS-42^T^ | 99.78 | *Rhizobium soli* DS-42 | 99.78 |
| *Rhizobium "subbaraonis"* | B4K015 | *Rhizobium* sp. PP-CC-3A-592 | 99.21 | *Rhizobium subbaraonis* JC85 | 97.39 |
| *Rhodobacter "ovatus"* | A1P081 | *Rhodobacter* *ovatus* JA234^T^ | 97.09 | *Rhodobacter ovatus* JA234 | 97.09 |
| *Roseomonas "aerophila"* | C2P008 | *Roseomonas* *aerophila* 7515T-07^T^ | 99.50 | *Roseomonas aerophila* 7515T-07 | 99.50 |
| *Roseomonas "elaeocarpi"* | B4P027b | *Roseomonas* *elaeocarpi* PN2^T^ | 99.35 | *Roseomonas elaeocarpi* PN2 | 99.35 |
| *Serinibacter "salmoneus"* | C3P045 | *Serinibacter* sp. PCH200 | 99.09 | *Serinibacter salmoneus* DSM 21801 | 97.35 |
| *Sphingobium "yanoikuyae"* 2 | A3K050 | *Sphingobium* *yanoikuyae* ATCC 51230^T^ | 99.71 | *Sphingobium yanoikuyae* ATCC 51230 | 99.71 |
| *Sphingobium yanoikuyae* | *Sphingobium yanoikuyae* DSM 7235 | - | - | - | - |
| *Sphingomonas "aerolata"* 1 | B3P008 | *Sphingomonas* sp. UV9 | 98.99 | *Sphingomonas aerolata* NW12 | 98.41 |
| *Sphingomonas "aerolata"* 2 | C2P058 | *Sphingomonas* sp. Leaf34 | 99.28 | *Sphingomonas aerolata* NW12 | 99.28 |
| *Sphingomonas "aerolata"* 3 | A3K041 | *Sphingomonas* sp. UV9 | 99.42 | *Sphingomonas aerolata* NW12 | 99.42 |
| *Sphingomonas "aquatilis"* 1 | D1K006 | *Sphingomonas* *aquatilis* JSS7^T^ | 97.84 | *Sphingomonas aquatilis* JSS7 | 97.84 |
| *Sphingomonas "aquatilis"* 2 | D1K002 | *Sphingomonas* *aquatilis* JSS7^T^ | 98.71 | *Sphingomonas aquatilis* JSS7 | 98.71 |
| *Sphingomonas "arantia"* 1 | C2K090 | *Sphingomonas* *arantia* 6P^T^ | 99.14 | *Sphingomonas arantia* 6P | 99.14 |
| *Sphingomonas "arantia"* 2 | B2K045 | *Sphingomonas* *arantia* 6P^T^ | 99.57 | *Sphingomonas arantia* 6P | 99.57 |
| *Sphingomonas "aurantiaca"* | B2K035a | *Sphingomonas* sp. Leaf198 | 99.50 | *Sphingomonas aurantiaca* MA101b | 99.35 |
| *Sphingomonas "cynarae"* 1 | A3K080 | *Sphingomonas* *cynarae* SPC-1^T^ | 98.70 | *Sphingomonas cynarae* SPC-1 | 98.70 |
| *Sphingomonas "cynarae"* 2 | C4P045 | *Sphingomonas* *cynarae* SPC-1^T^ | 99.06 | *Sphingomonas cynarae* SPC-1 | 99.06 |
| *Sphingomonas "cynarae"* 3 | B4P027a | *Sphingomonas* *cynarae* SPC-1^T^ | 99.13 | *Sphingomonas cynarae* SPC-1 | 99.13 |
| *Sphingomonas "cynarae"* 4 | C3K057 | *Sphingomonas* *cynarae* SPC-1^T^ | 99.93 | *Sphingomonas cynarae* SPC-1 | 99.93 |
| *Sphingomonas "echinoides"* | C1K071 | *Sphingomonas* *echinoides* ATCC 14820^T^ | 99.57 | *Sphingomonas echinoides* ATCC 14820 | 99.57 |
| *Sphingomonas "endophytica"* 1 | B3P001 | *Sphingomonas* sp. 5.2 | 99.93 | *Sphingomonas endophytica* YIM 65583 | 97.91 |
| *Sphingomonas "endophytica"* 2 | A1K072 | *Sphingomonas* *endophytica* YIM 65583^T^ | 99.11 | *Sphingomonas endophytica* YIM 65583 | 99.11 |
| *Sphingomonas faeni* 1 | *Sphingomonas faeni* DSM 14747^T^ | - | - | - | - |
| *Sphingomonas "faeni"* 2 | D2K059 | *Sphingomonas* sp. Leaf198 | 99.93 | *Sphingomonas faeni* MA-olki | 99.64 |
| *Sphingomonas "faeni"* 3 | D3P057 | *Sphingomonas* sp. Leaf198 | 100.00 | *Sphingomonas faeni* MA-olki | 99.71 |
| *Sphingomonas "faeni"* 4 | D2K033 | *Sphingomonas* sp. PP-CE-3A-406 | 99.78 | *Sphingomonas faeni* MA-olki | 99.42 |
| *Sphingomonas "faeni"* 5 | B2K066 | *Sphingomonas* sp. PP-CC-1A-547 | 99.57 | *Sphingomonas faeni* MA-olki | 99.50 |
| *Sphingomonas "faeni"* 6 | B4K025 | *Sphingomonas* sp. Leaf198 | 99.93 | *Sphingomonas faeni* MA-olki | 99.64 |
| *Sphingomonas "faeni"* 7 | D3K063 | *Sphingomonas* sp. Leaf198 | 100.00 | *Sphingomonas faeni* MA-olki | 99.71 |
| *Sphingomonas "faeni"* 8 | D3P024 | *Sphingomonas* sp. PP-CC-1A-547 | 99.86 | *Sphingomonas faeni* MA-olki | 99.78 |
| *Sphingomonas "faeni"* 9 | D4P045 | *Sphingomonas* sp. PP-CE-3A-406 | 99.71 | *Sphingomonas faeni* MA-olki | 99.49 |
| *Sphingomonas "faeni"* 10 | D1K025 | *Sphingomonas* sp. PP-CE-3A-406 | 99.86 | *Sphingomonas faeni* MA-olki | 99.49 |
| *Sphingomonas "faeni"* 11 | C4K034 | *Sphingomonas* sp. Leaf20 | 99.43 | *Sphingomonas faeni* MA-olki | 99.14 |
| *Sphingomonas "faeni"* 12 | D3K021 | *Sphingomonas* sp. PP-CE-3A-406 | 99.93 | *Sphingomonas faeni* MA-olki | 99.57 |
| *Sphingomonas "glacialis"* 1 | A1K076 | *Sphingomonas* *glacialis* C16y^T^ | 98.99 | *Sphingomonas glacialis* C16y | 98.99 |
| *Sphingomonas "glacialis"* 2 | D2K016 | *Sphingomonas* *glacialis* C16y^T^ | 98.85 | *Sphingomonas glacialis* C16y | 98.85 |
| *Sphingomonas "glacialis"* 3 | D1P008 | *Sphingomonas* *glacialis* C16y^T^ | 98.92 | *Sphingomonas glacialis* C16y | 98.92 |
| *Sphingomonas "glacialis"* 4 | C4K064 | *Sphingomonas* *glacialis* C16y^T^ | 99.00 | *Sphingomonas glacialis* C16y | 99.00 |
| *Sphingomonas "glacialis"* 5 | A4P035 | *Sphingomonas* *glacialis* C16y^T^ | 99.06 | *Sphingomonas glacialis* C16y | 99.06 |
| *Sphingomonas "melonis"* 1 | B2K049 | *Sphingomonas* *melonis* DAPP-PG 224^T^ | 97.76 | *Sphingomonas melonis* DAPP-PG 224 | 97.76 |
| *Sphingomonas "melonis"* 2 | A3K084 | *Sphingomonas* *melonis* DAPP-PG 224^T^ | 98.11 | *Sphingomonas melonis* DAPP-PG 224 | 98.11 |
| *Sphingomonas "melonis"* 3 | A3P068 | *Sphingomonas* *melonis* DAPP-PG 224^T^ | 99.43 | *Sphingomonas melonis* DAPP-PG 224 | 99.43 |
| *Sphingomonas "panacis"* | D1K009 | *Sphingomonas* *panacis* DCY99^T^ | 98.71 | *Sphingomonas panacis* DCY99 | 98.71 |
| *Sphingomonas "panni"* | A4K102 | *Sphingomonas* *panni* C52^T^ | 99.28 | *Sphingomonas panni* C52 | 99.28 |
| *Sphingomonas "taxi"* 1 | C4K017 | *Sphingomonas* *taxi* ATCC 55669^T^ | 99.64 | *Sphingomonas taxi* ATCC 55669 | 99.64 |
| *Sphingomonas "taxi"* 2 | A2P048b | *Sphingomonas* *taxi* ATCC 55669^T^ | 99.78 | *Sphingomonas taxi* ATCC 55669 | 99.78 |
| *Sphingomonas aurantiaca* | *Sphingomonas aurantiaca* DSM 14748^T^ | - | - | - | - |
| *Staphylococcus epidermidis* | *Staphylococcus epidermidis* 10547 | - | - | - | - |
| *Staphylococcus warneri* | *Staphylococcus warneri* DSM 30728 | - | - | - | - |
| *Stenotrophomonas rhizophila* | *Stenotrophomonas rhizophila* DSM 14405^T^ | - | - | - | - |
| *Variovorax "boronicumulans"* | C3K091 | *Variovorax* sp. EL159^T^ | 99.31 | *Variovorax boronicumulans* BAM-48 | 99.10 |
| *Variovorax "ginsengisoli"* | C1K098a | *Variovorax* *ginsengisoli* Gsoil 3165^T^ | 99.37 | *Variovorax ginsengisol*i Gsoil 3165 | 99.37 |
| *Variovorax "robiniae"* 1 | B4K076 | *Variovorax* sp. EPS | 99.03 | *Variovorax robiniae* UCM-G35 | 98.96 |
| *Variovorax "robiniae"* 2 | D3K069 | *Variovorax* sp. EPS | 99.09 | *Variovorax robiniae* UCM-G35 | 99.03 |
| *Variovorax "robiniae"* 3 | C4P084 | *Variovorax* sp. OV700 | 98.76 | *Variovorax robiniae* UCM-G35 | 98.62 |
| *Xanthomonas "campestris"* | B2P082 | *Xanthomonas* *campestris* ATCC 33913^T^ | 100.00 | *Xanthomonas campestris* ATCC 33913 | 100.00 |
| *Xanthomonas "cynarae"* | D3P049b | *Xanthomonas* *cynarae* CFBP 4188^T^ | 99.86 | *Xanthomonas cynarae* CFBP 4188 | 99.86 |

Similarity of the 16S rRNA gene of the reference isolates is listed based on a comparison using EzBioCloud. The species names presented in quotation marks refer to the most closely related species based on the 16S rRNA gene. For MALDI groups identified using the Bruker database, the respective reference strain is listed (score value > 2.3).

**Table S2:** Accession numbers or locus tags of the *Pseudomonas* reference species used to construct the MLSA tree.

| **Species** | **16S rRNA** | ***gyrB*** | ***rpoB*** | ***rpoD*** |
| --- | --- | --- | --- | --- |
| *P. graminis* DSM 11363^T^ (LMG 21661) | NR_026395 | SAMN05216197_105132 | SAMN05216197_12818 | SAMN05216197_107120 |
| *P. graminis* UASWS1507 | BBI10_00190 | BBI10_RS08665 | BBI10_RS10625 | BBI10_RS19855 |
| *P. graminis* WP8 | KU523561 | EC919_RS11660 | EC919_RS19245 | EC919_RS16280 |
| *P. lutea* DSM 17257 ^T^ (LMG 21974) | LT42_12565 | LT42_20900 | LT42_RS20885 | LT42_RS24165 |
| *P. rhizosphaerae* DSM 16299 ^T^ | LT40_21195 | LT40_16300 | LT40_18635 | LT40_12390 |
| *P. coleopterorum* LMG 28558 ^T^ | BLV18_RS21585 | BLV18_RS21115 | BLV18_RS18445 | BLV18_RS18730 |
| *P. straminea* JCM 2783 ^T^ | BM279_RS23015 | BM279_RS03820 | BM279_RS22940 | BM279_RS21500 |
| *P. flavescens* LMG 18387 ^T^ (DSM 12071) | SAMN05216588_1471 | SAMN05216588_103342 | SAMN05216588_11120 | SAMN05216588_108212 |
| *P. jessenii* BS3660 ^T^ (DSM 17150) | SAMN04490187_0006 | SAMN04490187_0934 | SAMN04490187_0326 | SAMN04490187_0403 |
| *P. extremaustralis* DSM 17835 ^T^ | SAMN05216591_2461 | SAMN05216591_4629 | SAMN05216591_5231 | SAMN05216591_5161 |
| *P. poae* BS2776^T^ (DSM 14936) | SAMN04490208_1318 | SAMN04490208_3218 | SAMN04490208_3904 | SAMN04490208_3828 |
| *P. trivialis* BS3111^T^ (DSM 14937) | SAMN04490205_0433 | SAMN04490205_1409 | SAMN04490205_0771 | SAMN04490205_0838 |
| *P. fluorescens* DSM 50090 ^T^ | Ga0125782_1146 | Ga0125782_113633 | Ga0125782_112322 | Ga0125782_11350 |
| *P. veronii* DSM 11311 ^T^ | Ga0113058_1741 | Ga0113058_11244 | Ga0113058_12417 | Ga0113058_12149 |
| *P. caspiana* FBF102^T^ | Ga0308613_13334 | Ga0308613_11387 | Ga0308613_10916 | Ga0308613_111113 |
| *P. cerasi* 58^T^ | Ga0175993_13598 | Ga0175993_134 | Ga0175993_134674 | Ga0175993_13523 |
| *P. syringae* pv. *syringae* LMG 1247^T^ | Ga0124773_10951 | Ga0124773_107859 | Ga0124773_103516 | Ga0124773_114031 |
| *P. ficuserectae* LMG 5694^T^ | Ga0124757_16065 | Ga0124757_101221 | Ga0124757_110238 | Ga0124757_13954 |
| *P. congelans* DSM 14939 ^T^ | BLR20_RS26300 | BLR20_RS23130 | BLR20_RS19080 | BLR20_RS21500 |

The 16S rRNA, *gyrB*, *rpoB* and *rpoD* genes were used in this order for the concatenated alignment.

**Table S3:** Antagonistic potential of selected isolates assessed by measuring the inhibition of *H. fraxineus* growth and the vitality test of the remaining fungal mycelium.

| Isolate | Taxonomic assignment | Growth inhibition rate in co-culture (%) ^a^ | | Vitality of the mycelium (% of the untreated control) | |
| --- | --- | --- | --- | --- | --- |
|  |  | P3 | HF23 | P3 | HF23 |
| C4K093c | *Achromobacter „denitrificans“* | 40.0 ^*^ | 12.3 | 98.4 | 81.5 |
| A4P130 | *Bacillus „velezensis“* | 50.6 ^*^ | 54.7 ^*^ | 96.3 | 101.7 |
| C4K066b | *Bacillus „tequilensis”* | 35.1 | 37.5 | 0.0 ^b^ | 63.9 |
| C4P040b | *Bacillus „cereus”* | 30.0 | 35.0 | 98.5 | 91.7 |
| A2K052 | *Curtobacterium „flaccumfaciens”* 2 | 28.4 | 22.9 | 0.0 | 87.5 |
| D4K112a | *Curtobacterium „flaccumfaciens”* 2 | 19.8 | 21.8 | 70.6 ^b^ | 103.7 |
| A2P064 | *Erwinia „billingiae”* | 36.3 | 37.5 | 0.0 | 88.2 |
| B2P022 | *Erwinia „billingiae”* | 22.5 | 22.7 | 0.0 | 98.6 |
| D4P109 | *Erwinia „billingiae”* | 40.0 ^*^ | 22.5 | 0.0 ^b^ | 0.0 ^b^ |
| A3K051 | *Erwinia „billingiae”* | 35.0 | 35.4 | 0.0 | 110.0 |
| A4K048b | *Erwinia „billingiae”* | 35.9 | 39.6 ^*^ | 0.0 | 75.0 |
| C1P082 | *Frigoribacterium „ faeni“* 5 | 18.2 | 20.7 | 0.0 | 97.3 |
| B3K063 | *Frigoribacterium „ faeni“* 5 | 44.4 ^*^ | 37.5 | 0.0 | 82.9 |
| D1P082 | *Luteimonas „aestuarii“* | 22.5 | 25.6 | 64.8 | 86.2 |
| C4P040a | *Luteimonas „aestuarii“* | 20.5 | 25.3 | 79.4 | 76.7 |
| D4P002 | *Luteimonas „aestuarii“* | 12.5 | 24.3 | 57.5 | 92.9 |
| D3K057 | *Methylobacterium „bullatum“* 1 | 26.7 | 3.8 | 95.9 | 112.9 |
| D3P076 | *Methylobacterium „bullatum“* 1 | 30.0 | 2.4 | 100.0 | 90.7 |
| C4K020 | *Methylobacterium „cerastii”* | 25.3 | 4.4 | 0.0 ^b^ | 94.4 |
| D3P082 | *Methylobacterium „goesingense”* 2 | 30.8 | 6.1 | 96.3 | 90.7 |
| C4K087b | *Methylobacterium „pseudosasicola”* 2 | 22.1 | 18.8 | 97.1 | 90.3 |
| D1K052 | *Methylobacterium „pseudosasicola”* 2 | 33.8 | 15.9 | 103.7 | 86.2 |
| D3P086b | *Methylobacterium „pseudosasicola”* 2 | 28.2 | 24.4 | 100.0 | 79.3 |
| C1P060 | *Microbacterium „hatanonis“* 2 | 39.5 ^*^ | 17.7 | 95.8 | 96.0 |
| D2K023 | *Novosphingobium „fluoreni“* 4 | 35.0 | 34.6 | 85.9 ^b^ | 89.8 |
| A4P030 | *Paenibacillus „lautus“* 2 | 35.4 | 28.9 | 57.5 | 94.5 |
| B3P057 | *Paenibacillus „lautus“* 2 | 36.0 | 4.9 | 100.0 | 83.3 |
| A4P026 | *Paenibacillus „lautus“* 2 | 44.4 ^*^ | 7.3 | 98.7 | 73.3 |
| B3P038 | *Paenibacillus „lautus“* 2 | 45.7 ^*^ | 7.3 | 67.6 ^b^ | 85.5 |
| B3P050 | *Paenibacillus „lautus“* 2 | 36.0 | 31.6 | 93.3 | 96.7 |
| B1P081 | *Pantoea „vagans“* | 43.0 ^*^ | 39.4 ^*^ | 0.0 | 94.2 |
| B3K066 | *Pantoea „vagans“* | 40.5 ^*^ | 47.5 ^*^ | 0.0 | 80.8 |
| A3K039 | *Pantoea „vagans“* | 45.0 ^*^ | 40.0 ^*^ | 0.0 | 90.7 |
| A2P019a | *Pantoea „vagans“* | 47.5 ^*^ | 47.0 ^*^ | 0.0 | 90.0 |
| C4P067 | *Pantoea „vagans“* | 24.7 | 24.4 | 98.6 | 94.5 |
| D3K037 | *Pantoea „vagans“* | 34.7 | 47.5 ^*^ | 27.4 ^b^ | 71.4 |
| B1P055 | *Pseudomonas „caspiana“* 3 | 41.0 ^*^ | 43.0 ^*^ | 0.0 | 86.8 |
| A2P018 | *Pseudomonas „caspiana“* 3 | 30.4 | 22.9 | 0.0 ^b^ | 90.0 ^b^ |
| A1P062 | *Pseudomonas „cerasi“* | 32.1 | 36.7 | 0.0 | 80.5 |
| A2P053 | *Pseudomonas „coleopterorum”* 2 | 33.8 | 24.2 | 0.0 | 92.3 |
| B2K010 | *Pseudomonas „coleopterorum”* 2 | 26.3 | 24.1 | 0.0 ^b^ | 63.3 |
| B2K013 | *Pseudomonas „coleopterorum”* 2 | 32.1 | 26.8 | 0.0 | 1.9 |
| B2K002a | *Pseudomonas „coleopterorum”* 2 | 28.8 | 35.0 | 0.0 ^b^ | 68.1 |
| D3P009a | *Pseudomonas „coleopterorum”* 2 | 21.0 | 24.4 | 97.3 | 102.6 |
| D3P095b | *Pseudomonas „coleopterorum”* 2 | 25.3 | 29.3 | 100.0 | 92.6 |
| D4P037 | *Pseudomonas „flavescens”* | 6.7 | 6.3 | 27.4 ^b^ | 92.9 |
| A3P029 | *Pseudomonas „graminis“* 1 | 32.5 | 7.4 | 0.0 ^b^ | 98.7 |
| A3P049 | *Pseudomonas „graminis“* 1 | 29.6 | 17.5 | 0.0 ^b^ | 87.2 |
| A4K088 | *Pseudomonas „graminis“* 1 | 25.0 | 2.5 | 87.7 | 97.3 |
| A3P013 | *Pseudomonas „graminis“* 1 | 22.2 | 2.5 | 95.0 | 87.8 |
| C1P044 | *Pseudomonas „graminis“* 1 | 6.7 | 26.8 | 64.0 | 55.6 |
| D3K026 | *Pseudomonas „graminis“* 1 | 34.7 | 6.3 | 102.7 | 84.3 |
| B3P024 | *Pseudomonas „graminis“* 2 | 23.5 | 19.3 | 65.8 | 96.4 |
| A4K089 | *Pseudomonas „extremaustralis”* | 31.3 | 21.5 | 0.0 ^b^ | 92.0 |
| A2P026 | *Pseudoxanthomonas „spadix“* | 35.0 | 15.7 | 91.5 | 84.4 |
| A2P086 | *Rahnella „victoriana“* | 33.3 | 26.2 | 0.0 | 97.4 |
| B3P075 | *Rhizobium „skierniewicense “* | 31.3 | 30.0 | 0.0 | 86.7 |
| C4K021 | *Rhizobium „skierniewicense “* | 36.3 | 27.5 | 0.0 ^b^ | 90.3 |
| B3P008 | *Sphingomonas „aerolata“* 1 | 36.4 | 32.9 | 0.0 ^b^ | 100.0 |
| D2K022 | *Sphingomonas „aurantiaca“* | 37.5 | 30.9 | 76.6 ^b^ | 95.2 |
| B2K034 | *Sphingomonas faeni* 1 | 29.2 | 31.3 | 93.9 | 92.5 |
| B3P014 | *Sphingomonas faeni* 1 | 25.9 | 31.7 | 66.7 | 100.0 |
| B4P041 | *Sphingomonas faeni* 1 | 36.3 | 21.3 | 0.0 | 94.2 |
| B3K005 | *Sphingomonas faeni* 1 | 38.7 ^*^ | 32.9 | 0.0 | 90.3 |
| C2K097 | *Sphingomonas faeni* 1 | 32.5 | 34.6 | 85.9 ^b^ | 92.2 |
| A4K001 | *Sphingomonas faeni* 1 | 35.1 | 23.1 | 97.0 | 93.2 |
| A4P019 | *Sphingomonas „faeni“* 3 | 32.1 | 28.6 | 0.0 | 100.0 |
| D4P108 | *Sphingomonas „taxi“* 1 | 22.5 | 35.0 | 98.6 | 100.0 |
| A4K048b | *Sphingomonas „taxi“* 2 | 27.0 | 24.6 | 98.5 | 100.0 |
| B4K076 | *Variovorax „robiniae“* 1 | 26.7 | 26.8 | 96.0 | 88.7 |
| B2P007 | *Xanthomonas „cynarae“* | 30.8 | 31.8 | 0.0 ^b^ | 86.7 |
| B3P052 | *Xanthomonas „cynarae“* | 36.1 | 28.8 | 79.4 ^b^ | 95.0 |
| B1P009 | *Xanthomonas „cynarae“* | 30.9 | 27.5 | 0.0 | 92.0 |
| B1P028 | *Xanthomonas „cynarae“* | 26.3 | 33.3 | 0.0 | 90.7 |
| A4P033 | *Xanthomonas „cynarae“* | 44.4 ^*^ | 28.9 | 0.0 | 81.0 |
| A4K091 | *Xanthomonas „cynarae“* | 37.3 ^*^ | 36.6 | 100.0 | 100.0 |
| C2P069 | *Xanthomonas „cynarae“* | 37.5 ^*^ | 27.2 | 0.0 ^b^ | 101.6 |
| D3P045 | *Xanthomonas „cynarae“* | 42.5 ^*^ | 33.3 | 42.2 ^b^ | 100.0 |

The 78 isolates were selected based on the first antagonistic screening and dereplication of the total isolates.
